# Unlocking the dual power of *Charybdis natator* shell: antiviral and larvicidal activities

**DOI:** 10.1186/s40643-025-00868-7

**Published:** 2025-04-04

**Authors:** Karnan Ramachandran, Senthil Bakthavatchalam, Shunmuga Vadivu Ramalingam, Ramachandran Vinayagam, Mukeshwaran Ramesh, Sukumaran Marimuthu, Zhi-Hong Wen, Chandramohan Govindasamy, Khalid M. Almutairi, Yi-Hao Lo

**Affiliations:** 1 PG and Research Department of Zoology, Rajah Serfoji Government College (Autonomous), Affiliated to Bharathidasan University, Thanjavur, Tamil Nadu 613 005 India; 2 Department of Chemistry, Faculty of Engineering and Technology, SRM Institute of Science and Technology, Ramapuram, Chennai, Tamil Nadu 600089 India; 3 Department of Biochemistry, SRM Dental College, Bharathi Salai, Ramapuram, Chennai, Tamil Nadu 600089 India; 4 Department of Biotechnology, College of Life and Applied Sciences, Yeungnam University, 280 Daehak-Ro, Gyeongsan, Gyeongsangbuk-do 38541 Republic of Korea; 5 ACS Medical College, Chennai, India; 6 National Museum of Marine Biology and Aquarium, Pingtung, 94450 Taiwan; 7 Department of Marine Biotechnology and Resources, National Sun Yat-Sen University, Kaohsiung, 80424 Taiwan; 8 Institute of Biopharmaceutical Sciences, National Sun Yat-sen University, Kaohsiung, 80424 Taiwan; 9 Department of Community Health Sciences, College of Applied Medical Sciences, King Saud University, P.O. Box 10219, 11433 Riyadh, Saudi Arabia; 10 Department of Family Medicine, Zuoying Armed Forces General Hospital, Kaohsiung, 81342 Taiwan; 11 Department of Nursing, Meiho University, Pingtung County, 91200 Taiwan

**Keywords:** Antiviral, Arbovirus vector, *Charybdis natator*, Zoo-virucide, Crab zoochemicals

## Abstract

This study investigates the in silico anti-arboviral potential of zoochemicals derived from the methanolic extract of *Charybdis natator* shell, alongside their larvicidal efficacy against *Aedes aegypti* 4th instar larvae. Through GC–MS analysis, 27 zoochemicals were identified, demonstrating promising in silico activity against molecular antiviral targets: DENV2 protease (PDB: 6MO1) for anti-dengue, RNA polymerase (PDB: 5U04) for anti-Zika, and nsP2 protease (PDB: 3TRK) for anti-chikungunya. A strong positive correlation (r = 0.726–0.889) in binding affinities (kcal/mol) suggests a consistent inhibitory mechanism across these targets. Furthermore, PASS analysis indicates higher probabilities of activity (Pa) for insecticidal properties compared to antiviral efficacy, highlighting their dual potential as larvicidal agents and antiviral candidates. The methanolic extract of *Charybdis natator* shell exhibited potent larvicidal activity against *Aedes aegypti* (LC₅₀ = 81.001 µg/mL) in a dose-dependent manner (R^2^ = 0.968). In silico analysis further elucidated its inhibitory action on key growth regulators of *A. aegypti,* underscoring its potential to disrupt larval development. These findings highlight the dual utility of *C. natator* shell extract in vector management and in mitigating the transmission of arboviral diseases such as Dengue, Zika, and Chikungunya. The extract's promise as an eco-friendly, cost-effective source for developing novel insecticidal and antiviral agents merits further exploration.

**Graphical Abstract:**

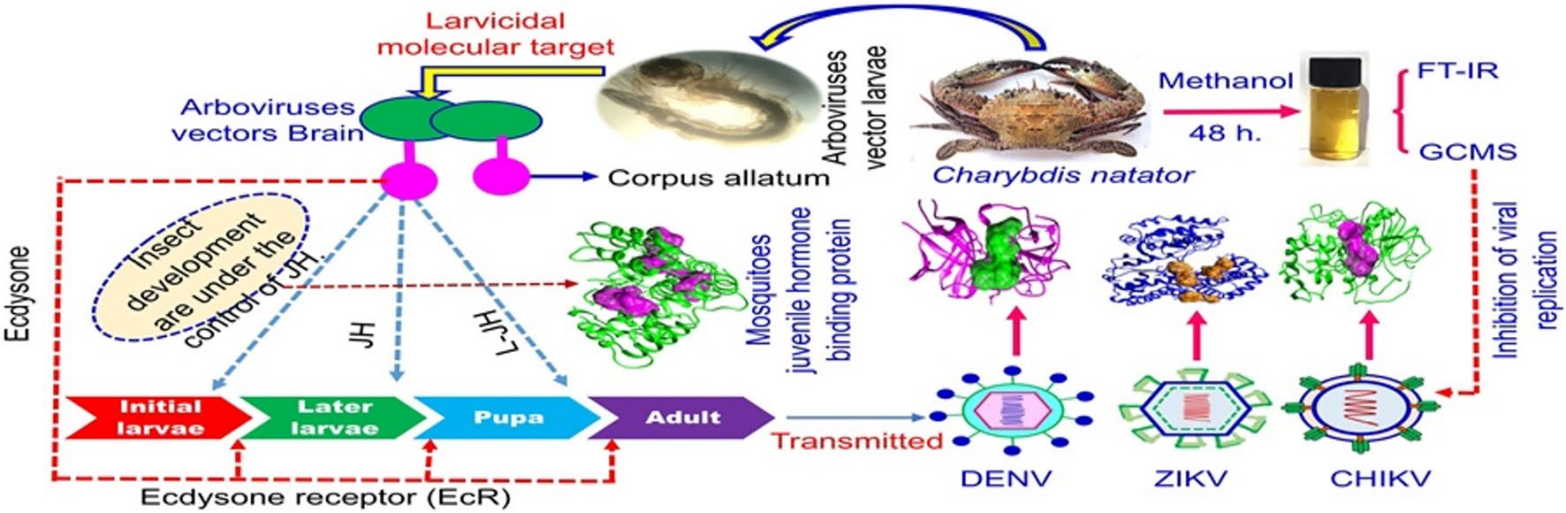

## Introduction

Arboviruses are responsible for numerous global outbreaks, leading to widespread infections such as Dengue (DENV), Zika (ZIKV), Chikungunya (CHIKV), Japanese Encephalitis (JE), and West Nile (WNV). These viral diseases contribute to millions of severe cases annually, with substantial morbidity and mortality, posing significant public health challenges worldwide (Puig-Torrents and Díez 2024; Dong and Dimopoulos 2021). Despite the growing threat of emerging arboviruses, substantial gaps remain in their control, as there are currently no specific antivirals and only a limited number of vaccines available (Goh et al. 2020). No licensed antiviral treatment exists for these infections, highlighting the urgent need for effective therapeutic options (Loaiza-Cano et al. 2021). Arboviruses are arthropod-borne viruses, primarily from the *Flaviviridae, Togaviridae*, and *Bunyaviridae* families, transmitted through primary arthropod vectors including mosquitoes, ticks, and sandflies (Chen et al. 2018; Simmonds et al. 2017; Weaver 2005). Arboviruses such as dengue and chikungunya are significant infectious diseases in tropical and subtropical regions, primarily transmitted by *Aedes* mosquitoes (Alagarasu et al. 2022). Outbreaks of chikungunya and dengue fever are chiefly linked to *Aedes aegypti* and *Aedes albopictus* (Aragão et al. 2019). *Aedes* species are the primary vectors for major arboviruses, and effective control of these mosquitoes plays a critical role in mitigating the spread of DENV, ZIKV, and CHIKV infections.

Marine invertebrates are widely recognized for their use as food and feed additives across the globe (Oliveira et al. 2019). These organisms, similar to their terrestrial counterparts, produce a diverse array of secondary metabolites (Eiken 2024; Izzati et al. 2021). Marine species such as sponges and sea urchins are particularly notable for synthesizing compounds that play roles in copper ion reduction and act as capping agents, exhibiting promising insecticidal properties (Karnan et al. 2023a, 2023d). The marine sponge *Hyattella intestinalis* is rich in alkaloids, including tryptamine, as identified by HPLC analysis (Karnan et al. 2023b). The chemical composition of *Lophozozymus incises* (Montagu's crab) was explored to better understand its biological properties and potential health benefits. Using GC–MS and HPLC–DAD, twenty-eight metabolites were identified in the ethanol extract, with astaxanthin (57%) and alanine (33%) being the predominant carotenoid and amino acid, respectively. Additionally, unsaturated fatty acids comprised 86.0% of the total fatty acids, highlighting their significant role in the organism's biochemical profile (Oliveira et al. 2019). The shells of mud crabs (*Scylla serrata*) contain bioactive compounds, such as astaxanthin, which may possess antioxidant properties (Karnila et al. 2021). In studies using the edible crab *Callinectes sapidus*, extracts from the hepatopancreas and hemolymph were assessed for in vitro cytotoxicity against human cell lines. These extracts demonstrated potent antiproliferative effects on HeLa cells, with IC_50_ values of 0.39 and 2.01 mg/mL, respectively. These findings suggest that *Callinectes sapidus* extracts hold potential for developing novel therapies for human cervical adenocarcinoma (Somaia et al. 2021).

Viral DNA and RNA polymerases are essential enzymes for viral replication. These enzymes have emerged as highly promising targets for the development of antiviral drugs (Peng et al. 2022). Among these, RNA-dependent RNA polymerase (RdRp) plays a crucial role in viral replication and is considered a prime target for antiviral drug development (Xu et al. 2022; Hasan et al. 2021). The NS2B-NS3 protease is a key enzyme in the replication of flaviviruses, including dengue virus. It has emerged as a promising target for antiviral drug development (Kühl et al. 2021; Adawara et al. 2021). Similarly, the cysteine protease domain of non-structural protein 2 (nsP2) is essential for the replication of chikungunya virus (CHIKV) and is considered a valuable target for antiviral drug development (Merten et al. 2024; Saha et al. 2018). Computational screening has identified DNV2 proteins, such as NS2B-NS3 protease, NS5 RdRp, and nsP2 protease, as potential targets for developing antiviral drugs against DENV, ZIKV, and CHIKV (Indu et al. 2022; Dantas et al. 2021; Adawara et al. 2020).

Marine organism also produces a diverse array of secondary metabolites known as zoochemicals. These compounds often exhibit potent biological activities, including antimicrobial, antiviral, and insecticidal properties. This study aims to discover novel bioactive compounds with larvicidal and antiviral properties from a marine source, *Charybdis natator* crab shells. This approach focuses on targeting both the vector and the virus itself, offering a comprehensive strategy for combating arboviral infections.

## Materials and methods

### Zoo-extract extraction and zoochemicals screening

**Fig. 1:**
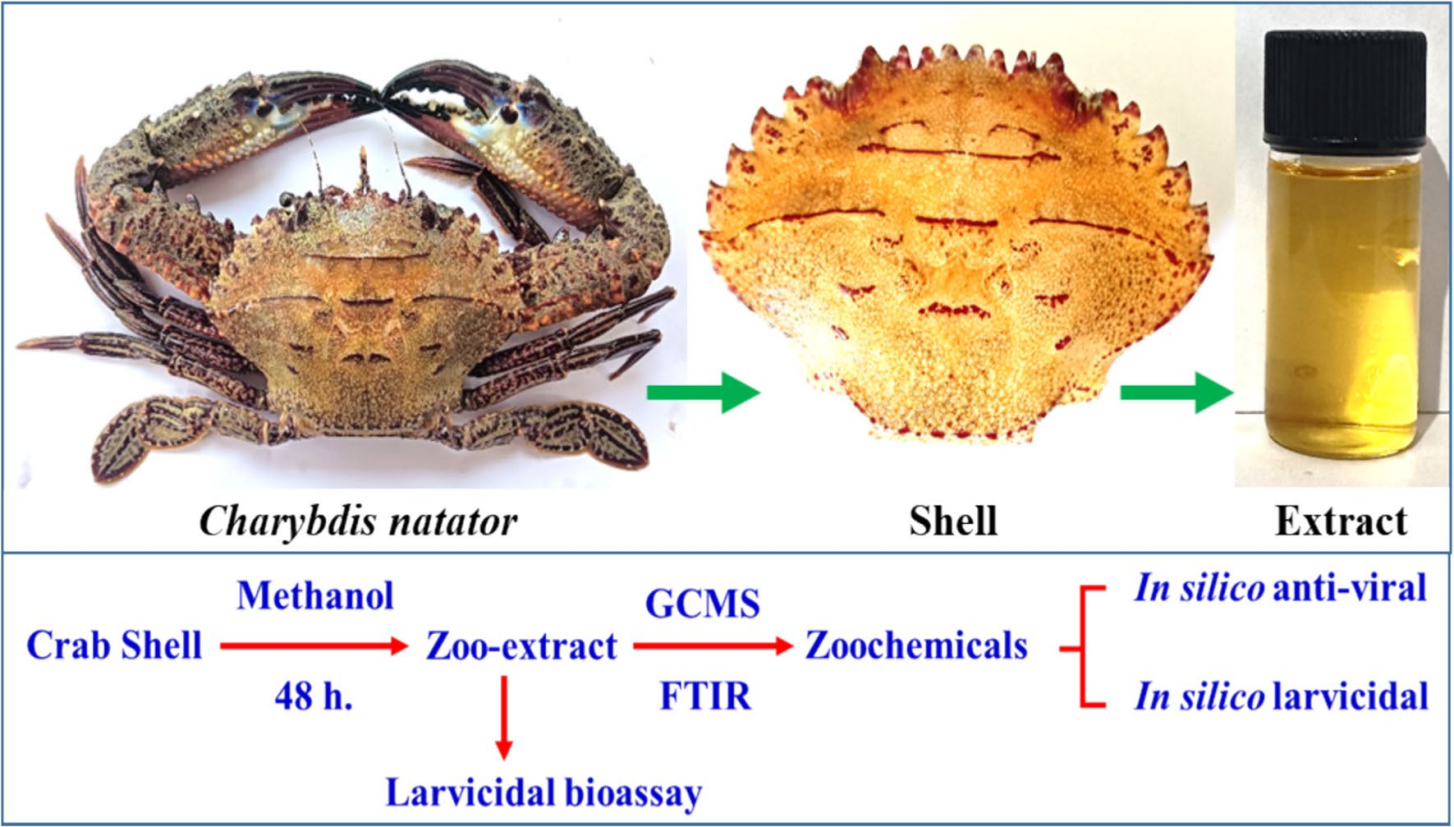
Methanolic extraction of *Charybdis natator* shell

Two kilograms of marine crab *Charybdis natator* were procured from the Thanjavur fish market, Tamil Nadu, India. The crab shells were subjected to methanolic extraction following the protocol of Galal-Khallaf et al. (2024), with minor modifications. The shells were carefully removed, air-dried at room temperature, and finely powdered using a mortar and pestle. A 20-g sample of the powdered shell was extracted with 100 mL of methanol over 48 h in a dark environment. Upon completion, the resulting red-colored extract was collected and stored. The remaining shell residue was re-extracted twice, yielding a colorless extract devoid of zoochemicals. The red-colored extracts were combined, concentrated under reduced pressure at 40 ºC, and prepared for subsequent zoochemical screening and larvicidal assays (Fig. 1).

GC–MS and FTIR techniques were employed for the preliminary zoochemical analysis of the methanolic extract from the crab shells. The components were identified by comparing their retention times with those in the NIST and Wiley databases linked to the GC–MS instrument. Identification was further validated by matching the detected compounds against entries in these reference libraries (Gomathi et al. 2015).

### ADMET prediction

Swiss ADME (http://www.swissadme.ch) and ProTox-3.0 (https://tox.charite.de/protox3/) web servers were utilized to predict the ADMET (absorption, distribution, and metabolism and toxicity) of the selected zoochemicals of *Charybdis natator* shell (Abdul-Hammed et al. 2021).

### PASS prediction

The zoochemicals from the *Charybdis natator* shell methanolic extract were analyzed for their antiviral and insecticidal potential using the Prediction of Activity Spectra for Substances (PASS) software (Filimonov et al. 2014; Jamkhande et al. 2014). As described by Khurana et al. (2011), the predicted activity of a compound is represented by the probable activity (Pa) and probable inactivity (Pi) values. According to Filimonov et al. (2014), these values are scored on a scale from 0.000 to 1.000, where Pa + Pi ≠ 1. If Pa > Pi, the compound is more likely to exhibit the predicted activity. A Pa value greater than 0.7 suggests a high probability of experimental confirmation, while values between 0.5 and 0.7 indicate a moderate chance. If Pa is less than 0.5, the likelihood of confirming the activity experimentally diminishes, though structural similarities to known pharmaceuticals may still be present.

### Molecular docking

The antiviral and insecticidal ligands from the *Charybdis natator* shell methanolic extract were identified through GC–MS analysis, and the corresponding zoochemicals were retrieved from the PubChem database. The selected antiviral targets included the 3D structures of dengue virus protease (PDB ID: 6MO1; Resolution: 3.00 Å), Zika virus NS5 RNA-dependent RNA polymerase (PDB ID: 5U04; Resolution: 1.90 Å), Chikungunya virus nsP2 protease (PDB ID: 3TRK; Resolution: 2.40 Å), and the larvicidal target, mosquito juvenile hormone-binding protein (PDB ID: 5V13; Resolution: 1.84 Å), all sourced from the PDB database. Prior to molecular docking, all water molecules and other ligands in the protein structures were removed. Molecular docking was performed using the PyRx tool (Version 0.8) with the AutoDock Vina algorithm, guided by scoring functions and grid dimensions (Trott and Olson 2010). Post-docking analyses of the ligand-target interactions were visualized using PyMOL (Version 2.3.2), BIOVIA Discovery Studio Visualizer (Version 2021), and UCSF Chimera (Version 1.14).

### Larvicidal activity of *Charybdis natator* shell methanolic extract

The *Aedes aegypti* (Linnaeus, 1762) mosquitoes used in the study were identified by David Pecor (2022) and maintained in a laboratory environment with a rearing medium of deionized water supplemented with glucose and yeast powder. The colony was kept under controlled conditions at 27 °C, with a relative humidity of 75 ± 5% and a 14 h photoperiod, following a standard rearing protocol with minor modifications (Soni and Dhiman 2020). The methanolic extract of *C. natator* shell, at concentrations of 50, 100, 150, 200, and 250 µg/mL was evaluated for its larvicidal activity against *Aedes aegypti* 4th instar larvae. The bioassay was conducted using the standard protocol outlined by the WHO (2005).

### Statistical analysis

All experiments were conducted in triplicate (n = 3), and the data were analyzed using IBM SPSS Version 20.0, with subsequent probit analysis. The LC_50_ and LC_90_ values, along with their 95% confidence intervals, were also calculated.

## Results and discussion

### Zoochemical screening and ADMET profile of *Charybdis natator* shell extract

**Fig. 2:**
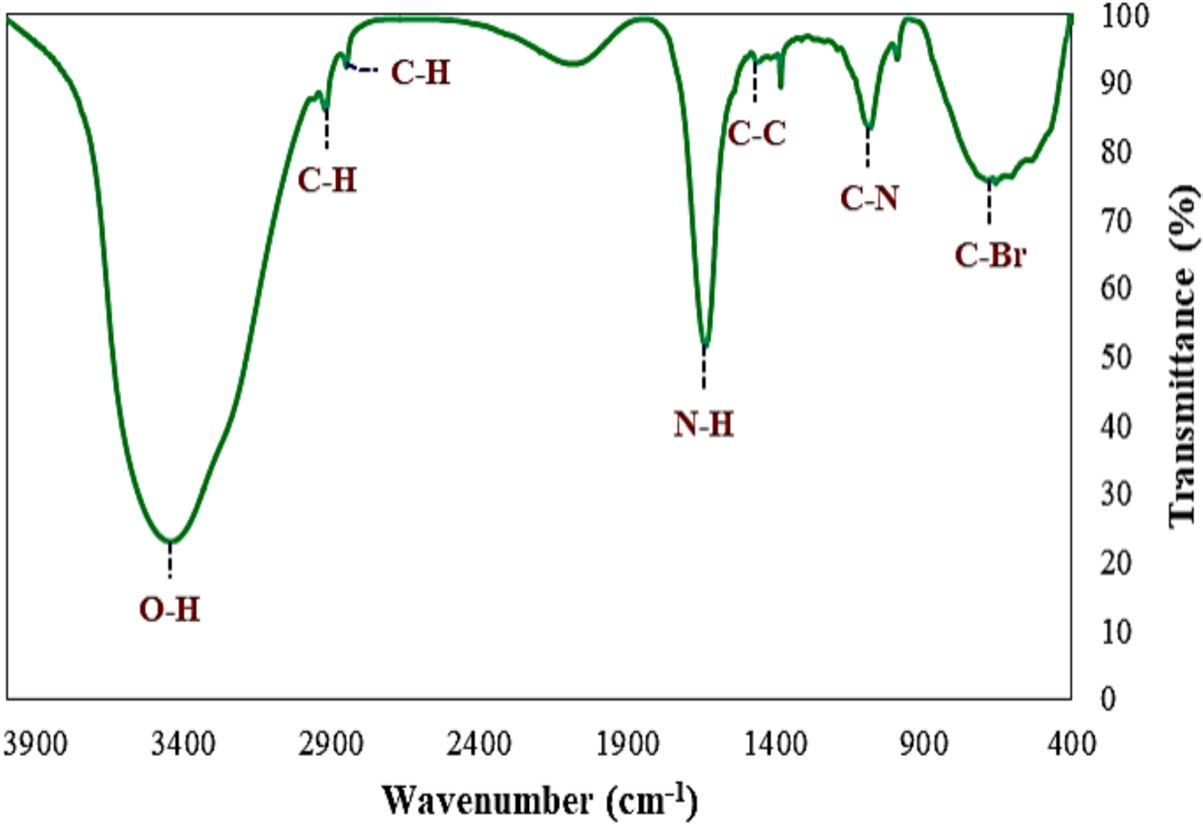
FTIR spectra of *Charybdis natator* shell methanolic extract

**Fig. 3:**
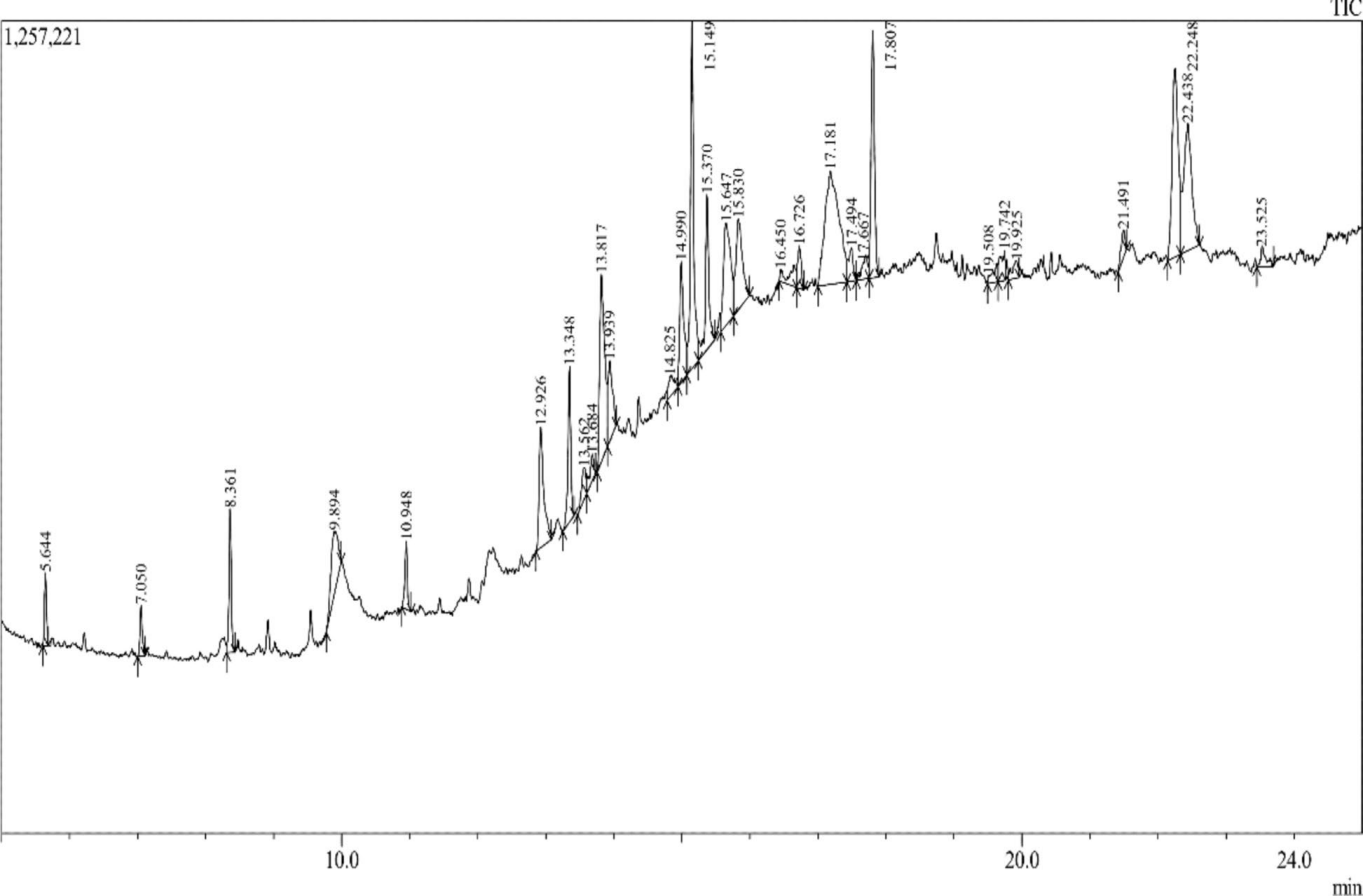
GC–MS chromotogram of *Charybdis natator* shell methanolic extract

Zoochemicals, akin to plant-derived phytochemicals, encompass bioactive secondary metabolites with diverse biological functions. Marine invertebrates, such as sea urchins and sponges, are prolific sources of these compounds (Karnan et al. 2023a, b, c, d). Methanolic extracts from *Charybdis natator* shells revealed functional groups characteristic of alcohols, phenols, alkanes, amines, aromatics, alkenes, and alkyl halides, confirming the presence of phenolic and alkaloid derivatives (Table 1, Fig. 2). Gas chromatography-mass spectrometry (GC–MS) analysis identified 27 distinct zoochemicals (Table 2 and Fig. 3), with major constituents including 9-Octadecenamide, (Z) (C_18_H_35_NO), Diisooctyl phthalate (C_24_H_38_O_4_), 9-Octadecenoic acid, methyl ester (C_19_H_36_O_2_), Hexadecane (C_16_H_34_), n-Hexadecanoic acid (C_16_H_32_O_2_), Nonadecanoic acid, methyl ester (C_20_H_40_O_2_) and 9-Octadecenoic acid, (E)- (C_18_H_34_O_2_). Other compounds were detected in lower quantities, underscoring the chemical complexity and potential bioactivity of the extract. Galal-Khallaf et al. (2024) identified a significant lipid composition in *Charybdis natator* shell methanolic extract, comprising 61% unsaturated fatty acids and 39% saturated fatty acids. Among the saturated fatty acids, palmitic acid (10%), stearic acid (6%), and 11-octadecenoic acid (14%) were predominant, as determined by GC–MS analysis. Similarly, methanolic extracts from the Nile crab (*Potamonautes niloticus*) shells revealed key constituents such as palmitic acid, oleic acid, and stearic acid (Galal-Khallaf et al. 2022). Strong evidence for the presence of *C. natator* shell extract includes n-hexadecanoic acid, 9-octadecenoic acid, and octadecanoic acid. Additional compounds identified in *C. natator* shell extracts include 1-heptatriacotanol, dotriacontane, ethyl iso-allocholate, tert-hexadecanethiol, 11-octadecenoic acid, hexadecanoic acid, and tetradecane, highlighting its diverse and bioactive chemical profile (Elshaarawy et al. 2023). Thus, 9-octadecenoic acid (C_18_H_34_O_2_) and n-hexadecanoic acid (C_16_H_32_O_2_) have been reported to exhibit insect-repellent and pesticidal properties (Natarajan et al. 2019; Hema et al. 2011). Tables 3, 4, 5 shows ADMET properties of identified *Charybdis natator* shell, with a significant role in biological activity (Tables 3, 4, 5). ADMET characteristics are important in the early phases of oral bioavailability studies and drug discovery (Abdul-Hammed et al. 2021; Guan et al. 2018).

**Table 1:** Functional group analysis of *Charybdis natator* shell methanolic extract, using FT-IR techniques

Wavelength of absorption (cm^−1^)	Mode of vibration	Functional group
3445.51	O–H stretch, H-bonded	Alcohols, Phenols
2922.96, 2851.67	C–H stretch	Alkanes
1637.41	N–H bend	1° amines
1465.64	C–C stretch	Aromatics
1384.39	C–H rock	Alkanes
1083.29	C–N stretch	Aliphatic amines
990.64	= C–H bend	Alkenes
657.74	C–Br stretch	Alkyl halides

**Table 2:** Zoochemicals screening of *Charybdis natator* shell methanolic extract, using GC–MS

Peak #	R. Time	Area %	Height %	M. weight (g/mol)	M. formula	Tentative identified zoochemicals
1	5.644	1.13	2.70	198	C_14_H_30_	Dodecane, 4,6-dimethyl
2	7.050	0.91	1.87	182	C_13_H_26_	1-Tridecene (CAS) n-Tridec-1-ene
3	8.361	2.44	5.28	240	C_17_H_36_	Heptadecane
4	9.894	3.40	2.28	222	C_12_H_14_O_4_	1,2-Benzenedicarboxylic acid, diethyl ester (CAS) Ethyl phthalate
5	10.948	1.27	2.51	170	C_12_H_26_	3,6-Dimethyldecane
6	12.926	4.36	4.44	376	C_23_H_36_O_4_	Phthalic acid, butyl undecyl ester
7	13.348	3.29	5.71	270	C_17_H_34_O_2_	Hexadecanoic acid, methyl ester (CAS) Methyl palmitate
8	13.562	1.05	1.16	–	–	Unidentified
9	13.684	0.83	0.97	243	C_12_H_10_FN_5_	1H-Purin-6-amine, [(2-fluorophenyl)methyl]
10	13.817	7.25	6.90	256	C_16_H_32_O_2_	n-Hexadecanoic acid
11	13.939	2.60	2.96	278	C_16_H_22_O_4_	Dibutyl phthalate
12	14.825	0.94	0.74	296	C_20_H_40_O	Phytol
13	14.990	3.74	4.44	284	C_19_H_40_O	n-Nonadecanol-1
14	15.149	8.44	12.79	296	C_19_H_36_O_2_	9-Octadecenoic acid, methyl ester (CAS) Methyl Octadec-9-Enoate
15	15.370	4.18	5.69	298	C_19_H_38_O_2_	Octadecanoic acid, methyl ester
16	15.647	5.51	3.78	282	C_18_H_34_O_2_	9-Octadecenoic acid, (E)-
17	15.830	4.58	3.33	284	C_18_H_36_O_2_	Octadecanoic acid
18	16.450	1.16	0.42	508	C_34_H_68_O_2_	Heptadecanoic acid, heptadecyl ester
19	16.726	1.11	1.57	–	–	Unidentified
20	17.181	12.16	4.17	281	C_18_H_35_NO	9-Octadecenamide, (Z)- (CAS) Oleoamide
21	17.494	1.17	1.23	324	C_21_H_40_O_2_	11-Eicosenoic acid, methyl ester
22	17.667	1.03	0.62	256	C_17_H_36_O	1-Heptadecanol
23	17.807	6.28	9.09	312	C_20_H_40_O_2_	Nonadecanoic acid, methyl ester
24	19.508	0.78	0.31	200	C_10_H_16_O_4_	Hexanedioic acid, 2-methyl-5-methylene-, dimethyl ester
25	19.742	1.10	1.10	–	–	Unidentified
26	19.925	0.78	0.63	254	C_17_H_34_O	4-Methyl-Z-4-hexadecen-1-ol
27	21.491	0.88	1.00	424	C_28_H_56_O_2_	Heptacosanoic acid, methyl ester
28	22.248	8.88	6.95	390	C_24_H_38_O_4_	Diisooctyl phthalate
29	22.438	7.99	4.68	226	C_16_H_34_	Hexadecane
30	23.525	0.75	0.68	198	C_14_H_30_	Tetradecane

**Table 3:** ADMET analysis of Zoochemicals of *Charybdis natator* shell methanolic extract

Parameters	Zoochemicals								
	1,2-Benzenedicarboxylic acid, diethyl ester	1-Heptadecanol	1H-Purin-6-amine, [(2-fluorophenyl)methyl]	1-Tridecene	3,6-Dimethyldecane	4-Methyl-Z-4-hexadecen-1-ol	9-Octadecenamide, (Z)	9-Octadecenoic acid, (E)	Phthalic acid, butyl undecyl ester
Physicochemical properties									
Formula	C_12_H_14_O_4_	C_17_H_36_O	C_12_H_10_FN_5_	C_13_H_26_	C_12_H_26_	C_17_H_34_O	C_18_H_35_NO	C_18_H_34_O_2_	C_23_H_36_O_4_
Molecular weight (g/mol)	222.24	256.47	243.24	182.35	170.33	254.45	281.48	282.46	376.53
Num. heavy atoms	16	18	18	13	12	18	20	20	27
Num. arom. heavy atoms	6	0	15	0	0	0	0	0	6
Fraction Csp3	0.33	1.00	0.08	0.85	1.00	0.88	0.83	0.83	0.65
Num. rotatable bonds	6	15	3	10	7	13	15	15	17
Num. H-bond acceptors	4	1	4	0	0	1	1	2	4
Num. H-bond donors	0	1	2	0	0	1	1	1	0
Molar Refractivity	58.61	84.99	65.43	64.13	59.80	84.52	91.07	89.94	111.49
TPSA (Å^2^)	52.60	20.23	66.49	0.00	0.00	20.23	43.09	37.30	52.60
Lipophilicity									
Log *P*_o/w_ (iLOGP)	2.26	4.70	1.32	3.66	3.60	4.43	4.22	4.27	5.21
Log *P*_o/w_ (XLOGP3)	2.42	7.65	2.01	7.32	6.14	7.17	6.99	7.64	6.34
Log *P*_o/w_ (WLOGP)	2.04	5.85	2.18	5.09	4.64	5.63	5.51	6.11	6.33
Log *P*_o/w_ (MLOGP)	2.39	4.70	1.60	5.52	5.40	4.55	4.16	4.57	5.03
Log *P*_o/w_ (SILICOS-IT)	2.37	6.09	2.42	4.87	4.10	5.76	5.71	5.95	6.89
Consensus Log *P*_o/w_	2.30	5.80	1.91	5.29	4.77	5.51	5.32	5.71	5.96
Water solubility									
Log *S* (ESOL)	−2.62	−5.26	−3.03	−4.92	−4.30	−5.08	−5.00	−5.41	−5.21
Solubility	5.28e-01 mg/ml	1.41e-03 mg/ml	2.25e-01 mg/ml	2.18e-03 mg/ml	8.49e-03 mg/ml	2.13e-03 mg/ml	2.82e-03 mg/ml	1.09e-03 mg/ml	2.32e-03 mg/ml
Class	Soluble	Moderately soluble	Soluble	Moderately soluble	Moderately soluble	Moderately soluble	Moderately soluble	Moderately soluble	Moderately soluble
Log *S* (Ali)	−3.17	−7.91	−3.03	−7.15	−5.92	−7.42	−7.71	−8.26	−7.23
Solubility	1.51e-01 mg/ml	3.12e-06 mg/ml	2.25e-01 mg/ml	1.30e-05 mg/ml	2.04e-04 mg/ml	9.76e-06 mg/ml	5.49e-06 mg/ml	1.54e-06 mg/ml	2.19e-05 mg/ml
Class	Soluble	Poorly soluble	Soluble	Poorly soluble	Moderately soluble	Poorly soluble	Poorly soluble	Poorly soluble	Poorly soluble
Log *S* (SILICOS-IT)	−3.37	−6.17	−5.42	−4.76	−3.95	−5.44	−5.61	−5.39	−7.75
Solubility	9.55e-02 mg/ml	1.72e-04 mg/ml	9.14e-04 mg/ml	3.16e-03 mg/ml	1.92e-02 mg/ml	9.32e-04 mg/ml	6.89e-04 mg/ml	1.14e-03 mg/ml	6.65e-06 mg/ml
Class	Soluble	Poorly soluble	Moderately soluble	Moderately soluble	Soluble	Moderately soluble	Moderately soluble	Moderately soluble	Poorly soluble
Pharmacokinetics									
GI absorption	High	High	High	Low	Low	High	High	High	High
BBB permeant	Yes	No	Yes	No	No	Yes	Yes	No	No
P-gp substrate	No	No	Yes	No	No	No	No	No	No
CYP1A2 inhibitor	Yes	Yes	Yes	Yes	No	Yes	Yes	Yes	No
CYP2C19 inhibitor	No	No	No	No	No	No	No	No	No
CYP2C9 inhibitor	No	No	No	No	Yes	No	Yes	Yes	No
CYP2D6 inhibitor	No	No	No	No	No	No	No	No	No
CYP3A4 inhibitor	No	No	No	No	No	No	No	No	Yes
Log *K*_p_ (skin permeation)	−5.94 cm/s	−2.43 cm/s	−6.36 cm/s	−2.22 cm/s	−2.98 cm/s	−2.76 cm/s	−3.05 cm/s	−2.60 cm/s	−4.10 cm/s
Druglikeness									
Lipinski	Yes; 0 violation	Yes; 1 violation: MLOGP > 4.15	Yes; 0 violation	Yes; 1 violation: MLOGP > 4.15	Yes; 1 violation: MLOGP > 4.15	Yes; 1 violation: MLOGP > 4.15	Yes; 1 violation: MLOGP > 4.15	Yes; 1 violation: MLOGP > 4.15	Yes; 1 violation: MLOGP > 4.15
Ghose	Yes	No; 1 violation: WLOGP > 5.6	Yes	Yes	Yes	No; 1 violation: WLOGP > 5.6	Yes	No; 1 violation: WLOGP > 5.6	No; 1 violation: WLOGP > 5.6
Veber	Yes	No; 1 violation: Rotors > 10	Yes	Yes	Yes	No; 1 violation: Rotors > 10	No; 1 violation: Rotors > 10	No; 1 violation: Rotors > 10	No; 1 violation: Rotors > 10
Egan	Yes	Yes	Yes	Yes	Yes	Yes	Yes	No; 1 violation: WLOGP > 5.88	No; 1 violation: WLOGP > 5.88
Muegge	Yes	No; 2 violations: XLOGP3 > 5, Heteroatoms < 2	Yes	No; 3 violations: MW < 200, XLOGP3 > 5, Heteroatoms < 2	No; 3 violations: MW < 200, XLOGP3 > 5, Heteroatoms < 2	No; 2 violations: XLOGP3 > 5, Heteroatoms < 2	No; 1 violation: XLOGP3 > 5	No; 1 violation: XLOGP3 > 5	No; 2 violations: XLOGP3 > 5, Rotors > 15
Bioavailability score	0.55	0.55	0.55	0.55	0.55	0.55	0.55	0.85	0.55
Medicinal chemistry									
PAINS	0 alert	0 alert	0 alert	0 alert	0 alert	0 alert	0 alert	0 alert	0 alert
Brenk	1 alert: more_than_2_esters	0 alert	0 alert	1 alert: isolated_alkene	0 alert	1 alert: isolated_alkene	1 alert: isolated_alkene	1 alert: isolated_alkene	1 alert: more than 2 esters
Leadlikeness	No; 1 violation: MW < 250	No; 2 violations: Rotors > 7, XLOGP3 > 3.5	No; 1 violation: MW < 250	No; 3 violations: MW < 250, Rotors > 7, XLOGP3 > 3.5	No; 2 violations: MW < 250, XLOGP3 > 3.5	No; 2 violations: Rotors > 7, XLOGP3 > 3.5	No; 2 violations: Rotors > 7, XLOGP3 > 3.5	No; 2 violations: Rotors > 7, XLOGP3 > 3.5	No; 3 violations: MW > 350, Rotors > 7, XLOGP3 > 3.5
Synthetic accessibility	1.93	2.40	2.03	2.27	2.54	3.23	2.97	3.07	3.29
Toxicity (prediction/probability)									
LD50 (mg/kg)	6172	1000	500	5050	750	4300	750	48	1340
Hepatotoxicity	Inactive/0.77	Inactive/0.90	Inactive/0.69	Inactive/0.75	Inactive/0.75	Inactive/0.83	Inactive/0.82	Inactive/0.55	Inactive/0.84
Neurotoxicity	Inactive/0.81	Inactive/0.90	Active/0.78	Inactive/0.58	Inactive/0.66	Inactive/0.80	Active/0.52	Inactive/0.91	Inactive/0.82
Nephrotoxicity	Active/0.60	Inactive/0.67	Inactive/0.79	Inactive/0.87	Inactive/0.86	Inactive/0.69	Inactive/0.77	Inactive/0.55	Active/0.56
Respiratory toxicity	Inactive/0.97	Inactive/0.93	Active/0.76	Inactive/0.96	Active/0.85	Inactive/0.91	Inactive/0.87	Inactive/0.84	Inactive/0.99
Cardiotoxicity	Inactive/0.88	Inactive/1.0	Inactive/0.85	Inactive/0.79	Inactive/0.86	Inactive/0.83	Inactive/0.57	Inactive/0.99	Inactive/0.79

**Table 4:** ADMET analysis of Zoochemicals of *Charybdis natator* shell methanolic extract

Parameters	Zoochemicals								
	9-Octadecenoic acid, methyl ester	11-Eicosenoic acid, methyl ester	Dibutyl phthalate	Diisooctyl phthalate	Dodecane, 4,6-dimethyl	Heptacosanoic acid, methyl ester	Heptadecane	Heptadecanoic acid, heptadecyl ester	Phytol
Physicochemical properties									
Formula	C_19_H_36_O_2_	C_21_H_40_O_2_	C_16_H_22_O_4_	C_24_H_38_O_4_	C_14_H_30_	C_28_H_56_O_2_	C_17_H_36_	C_34_H_68_O_2_	C_20_H_40_O
Molecular weight (g/mol)	296.49	324.54	278.34	390.56	198.39	424.74	240.47	508.90	296.53
Num. heavy atoms	21	23	20	28	14	30	17	36	21
Num. arom. heavy atoms	0	0	6	6	0	0	0	0	0
Fraction Csp3	0.84	0.86	0.50	0.67	1.00	0.96	1.00	0.97	0.90
Num. rotatable bonds	16	18	10	16	9	26	14	32	13
Num. H-bond acceptors	2	2	4	4	0	2	0	2	1
Num. H-bond donors	0	0	0	0	0	0	0	0	1
Molar refractivity	94.26	103.87	77.84	116.30	69.41	138.00	83.83	166.84	98.94
TPSA (Å^2^)	26.30	26.30	52.60	52.60	0.00	26.30	0.00	26.30	20.23
Lipophilicity									
Log *P*_o/w_ (iLOGP)	4.75	5.24	2.97	5.42	3.95	7.28	4.91	8.68	4.85
Log *P*_o/w_ (XLOGP3)	7.45	8.24	4.50	8.41	7.22	12.91	8.82	16.26	8.19
Log *P*_o/w_ (WLOGP)	6.20	6.98	3.60	6.43	5.42	9.93	6.88	12.27	6.36
Log *P*_o/w_ (MLOGP)	4.80	5.25	3.43	5.24	5.93	6.81	6.68	7.95	5.25
Log *P*_o/w_ (SILICOS-IT)	6.54	7.42	3.97	6.98	4.98	10.69	6.65	13.34	6.57
Consensus Log *P*_o/w_	5.95	6.63	3.69	6.50	5.50	9.52	6.79	11.70	6.25
Water solubility									
Log *S* (ESOL)	−5.32	−5.86	−3.96	−6.66	−5.02	−8.89	−5.96	−11.13	−5.98
Solubility	1.43e-03 mg/ml	4.53e-04 mg/ml	3.03e-02 mg/ml	8.50e-05 mg/ml	1.87e-03 mg/ml	5.46e-07 mg/ml	2.62e-04 mg/ml	3.80e-09 mg/ml	3.10e-04 mg/ml
Class	Moderately soluble	Moderately soluble	Soluble	Poorly soluble	Moderately soluble	Poorly soluble	Moderately soluble	Insoluble	Moderately soluble
Log *S* (Ali)	−7.83	−8.65	−5.33	−9.38	−7.04	−13.50	−8.70	−16.98	−8.47
Solubility	4.34e-06 mg/ml	7.20e-07 mg/ml	1.32e-03 mg/ml	1.62e-07 mg/ml	1.80e-05 mg/ml	1.34e-11 mg/ml	4.76e-07 mg/ml	5.37e-15 mg/ml	9.94e-07 mg/ml
Class	Poorly soluble	Poorly soluble	Moderately soluble	Poorly soluble	Poorly soluble	Insoluble	Poorly soluble	Insoluble	Poorly soluble
Log *S* (SILICOS-IT)	−6.09	−6.89	−4.98	−7.40	−4.77	−10.36	−6.73	−12.70	−5.51
Solubility	2.40e-04 mg/ml	4.22e-05 mg/ml	2.93e-03 mg/ml	1.56e-05 mg/ml	3.38e-03 mg/ml	1.85e-08 mg/ml	4.45e-05 mg/ml	1.01e-10 mg/ml	9.06e-04 mg/ml
Class	Poorly soluble	Poorly soluble	Moderately soluble	Poorly soluble	Moderately soluble	Insoluble	Poorly soluble	Insoluble	Moderately soluble
Pharmacokinetics									
GI absorption	High	Low	High	High	Low	Low	Low	Low	Low
BBB permeant	No	No	Yes	No	No	No	No	No	No
P-gp substrate	No	No	No	No	No	Yes	No	Yes	Yes
CYP1A2 inhibitor	Yes	Yes	Yes	No	No	No	Yes	No	No
CYP2C19 inhibitor	No	No	Yes	No	No	No	No	No	No
CYP2C9 inhibitor	No	No	No	No	Yes	No	No	No	Yes
CYP2D6 inhibitor	No	No	No	No	No	No	No	No	No
CYP3A4 inhibitor	No	No	No	No	No	No	No	No	No
Log *K*_p_ (skin permeation)	−2.82 cm/s	−2.43 cm/s	−4.80 cm/s	−2.71 cm/s	−2.38 cm/s	0.28 cm/s	−1.50 cm/s	2.14 cm/s	−2.29 cm/s
Druglikeness									
Lipinski	Yes; 1 violation: MLOGP > 4.15	Yes; 1 violation: MLOGP > 4.15	Yes; 0 violation	Yes; 1 violation: MLOGP > 4.15	Yes; 1 violation: MLOGP > 4.15	Yes; 1 violation: MLOGP > 4.15	Yes; 1 violation: MLOGP > 4.15	No; 2 violations: MW > 500, MLOGP > 4.15	Yes; 1 violation: MLOGP > 4.15
Ghose	No; 1 violation: WLOGP > 5.6	No; 1 violation: WLOGP > 5.6	Yes	No; 1 violation: WLOGP > 5.6	Yes	No; 3 violations: WLOGP > 5.6, MR > 130, #atoms > 70	No; 1 violation: WLOGP > 5.6	No; 4 violations: MW > 480, WLOGP > 5.6, MR > 130, #atoms > 70	No; 1 violation: WLOGP > 5.6
Veber	No; 1 violation: Rotors > 10	No; 1 violation: Rotors > 10	Yes	No; 1 violation: Rotors > 10	Yes	No; 1 violation: Rotors > 10	No; 1 violation: Rotors > 10	No; 1 violation: Rotors > 10	No; 1 violation: Rotors > 10
Egan	No; 1 violation: WLOGP > 5.88	No; 1 violation: WLOGP > 5.88	Yes	No; 1 violation: WLOGP > 5.88	Yes	No; 1 violation: WLOGP > 5.88	No; 1 violation: WLOGP > 5.88	No; 1 violation: WLOGP > 5.88	No; 1 violation: WLOGP > 5.88
Muegge	No; 2 violations: XLOGP3 > 5, Rotors > 15	No; 2 violations: XLOGP3 > 5, Rotors > 15	Yes	No; 2 violations: XLOGP3 > 5, Rotors > 15	No; 3 violations: MW < 200, XLOGP3 > 5, Heteroatoms < 2	No; 2 violations: XLOGP3 > 5, Rotors > 15	No; 2 violations: XLOGP3 > 5, Heteroatoms < 2	No; 2 violations: XLOGP3 > 5, Rotors > 15	No; 2 violations: XLOGP3 > 5, Heteroatoms < 2
Bioavailability score	0.55	0.55	0.55	0.55	0.55	0.55	0.55	0.17	0.55
Medicinal chemistry									
PAINS	0 alert	0 alert	0 alert	0 alert	0 alert	0 alert	0 alert	0 alert	0 alert
Brenk	1 alert: isolated_alkene	1 alert: isolated_alkene	1 alert: more than 2 esters	1 alert: more than 2 esters	0 alert	0 alert	0 alert	0 alert	1 alert: isolated_alkene
Leadlikeness	No; 2 violations: Rotors > 7, XLOGP3 > 3.5	No; 2 violations: Rotors > 7, XLOGP3 > 3.5	No; 2 violations: Rotors > 7, XLOGP3 > 3.5	No; 3 violations: MW > 350, Rotors > 7, XLOGP3 > 3.5	No; 3 violations: MW < 250, Rotors > 7, XLOGP3 > 3.5	No; 3 violations: MW > 350, Rotors > 7, XLOGP3 > 3.5	No; 3 violations: MW < 250, Rotors > 7, XLOGP3 > 3.5	No; 3 violations: MW > 350, Rotors > 7, XLOGP3 > 3.5	No; 2 violations: Rotors > 7, XLOGP3 > 3.5
Synthetic accessibility	3.16	3.39	2.41	3.41	2.76	3.85	2.38	4.77	4.30
Toxicity (Prediction/ Probability)									
LD50 (mg/kg)	3000	3000	3474	1340	750	5000	750	5000	5000
Hepatotoxicity	Inactive/0.59	Inactive/0.59	Inactive/0.71	Inactive/0.79	Inactive/0.75	Inactive/0.58	Inactive/0.74	Inactive/0.76	Inactive/0.79
Neurotoxicity	Inactive/0.82	Inactive/0.82	Inactive/0.81	Inactive/0.84	Inactive/0.66	Inactive/0.80	Inactive/0.66	Inactive/0.87	Inactive/0.78
Nephrotoxicity	Inactive/0.60	Inactive/0.60	Active/0.56	Active/0.54	Inactive/0.86	Inactive/0.59	Inactive/0.87	Inactive/0.57	Inactive/0.74
Respiratory toxicity	Inactive/0.97	Inactive/0.97	Inactive/0.99	Inactive/0.99	Active/0.85	Inactive/0.98	Inactive/1.0	Inactive/0.99	Inactive/0.81
Cardiotoxicity	Inactive/0.98	Inactive/0.98	Inactive/0.84	Inactive/0.69	Inactive/0.86	Inactive/0.99	Inactive/0.83	Inactive 0.79	Inactive/0.84

**Table 5:** ADMET analysis of Zoochemicals of *Charybdis natator* shell methanolic extract

Parameters	Zoochemicals								
	Hexadecane	Hexadecanoic acid, methyl ester	Hexanedioic acid, 2-methyl-5-methylene-, dimethyl ester	n-Hexadecanoic acid	n-Nonadecanol-1	Nonadecanoic acid, methyl ester	Octadecanoic acid, methyl ester	Octadecanoic acid	Tetradecane
Physicochemical properties									
Formula	C_16_H_34_	C_17_H_34_O_2_	C_10_H_16_O_4_	C_16_H_32_O_2_	C_19_H_40_O	C_20_H_40_O_2_	C_19_H_38_O_2_	C_18_H_36_O_2_	C_14_H_30_
Molecular weight (g/mol)	226.44	270.45	200.23	256.42	284.52	312.53	298.50	284.48	198.39
Num. heavy atoms	16	19	14	18	20	22	21	20	14
Num. arom. heavy atoms	0	0	0	0	0	0	0	0	0
Fraction Csp3	1.00	0.94	0.60	0.94	1.00	0.95	0.95	0.94	1.00
Num. rotatable bonds	13	15	7	14	17	18	17	16	11
Num. H-bond acceptors	0	2	4	2	1	2	2	2	0
Num. H-bond donors	0	0	0	1	1	0	0	1	0
Molar refractivity	79.03	85.12	52.28	80.80	94.61	99.54	94.73	90.41	69.41
TPSA (Å^2^)	0.00	26.30	52.6	37.30	20.23	26.30	26.30	37.30	0.00
Lipophilicity									
Log *P*_o/w_ (iLOGP)	4.67	4.41	2.81	3.85	4.98	5.38	4.81	4.30	4.23
Log *P*_o/w_ (XLOGP3)	8.28	7.38	1.73	7.17	8.74	8.76	8.35	8.23	7.20
Log *P*_o/w_ (WLOGP)	6.49	5.64	1.30	5.55	6.63	6.81	6.42	6.33	5.71
Log *P*_o/w_ (MLOGP)	6.44	4.44	1.46	4.19	5.17	5.13	4.91	4.67	5.93
Log *P*_o/w_ (SILICOS-IT)	6.21	5.84	1.67	5.25	6.98	7.16	6.72	6.13	5.33
Consensus Log *P*_o/w_	6.42	5.54	1.79	5.20	6.50	6.65	6.24	5.93	5.68
Water solubility									
Log *S* (ESOL)	−5.60	−5.18	−1.71	−5.02	−5.99	−6.11	−5.83	−5.73	−4.88
Solubility	5.66e-04 mg/ml	1.80e-03 mg/ml	3.91e + 00 mg/ml	2.43e-03 mg/ml	2.92e-04 mg/ml	2.43e-04 mg/ml	4.42e-04 mg/ml	5.26e-04 mg/ml	2.62e-03 mg/ml
Class	Moderately soluble	Moderately soluble	Very soluble	Moderately soluble	Moderately soluble	Poorly soluble	Moderately soluble	Moderately soluble	Moderately soluble
Log *S* (Ali)	−8.14	−7.76	−2.45	−7.77	−9.05	−9.19	−8.77	−8.87	−7.02
Solubility	1.63e-06 mg/ml	4.68e-06 mg/ml	7.09e-01 mg/ml	4.31e-06 mg/ml	2.56e-07 mg/ml	2.00e-07 mg/ml	5.09e-07 mg/ml	3.80e-07 mg/ml	1.88e-05 mg/ml
Class	Poorly soluble	Poorly soluble	Soluble	Poorly soluble	Poorly soluble	Poorly soluble	Poorly soluble	Poorly soluble	Poorly soluble
Log *S* (SILICOS-IT)	−6.33	−6.01	−1.73	−5.31	−6.97	−7.21	−6.81	−6.11	−5.52
Solubility	1.06e-04 mg/ml	2.64e-04 mg/ml	3.73e + 00 mg/ml	1.25e-03 mg/ml	3.02e-05 mg/ml	1.94e-05 mg/ml	4.62e-05 mg/ml	2.19e-04 mg/ml	6.05e-04 mg/ml
Class	Poorly soluble	Poorly soluble	Soluble	Moderately soluble	Poorly soluble	Poorly soluble	Poorly soluble	Poorly soluble	Moderately soluble
Pharmacokinetics									
GI absorption	Low	High	High	High	Low	Low	High	High	Low
BBB permeant	No	Yes	Yes	Yes	No	No	No	No	No
P-gp substrate	No	No	No	No	No	No	No	No	No
CYP1A2 inhibitor	Yes	Yes	No	Yes	Yes	Yes	Yes	Yes	Yes
CYP2C19 inhibitor	No	No	No	No	No	No	No	No	No
CYP2C9 inhibitor	No	No	No	Yes	No	No	No	No	No
CYP2D6 inhibitor	No	No	No	No	No	No	No	No	No
CYP3A4 inhibitor	No	No	No	No	No	No	No	No	No
Log *K*_p_ (skin permeation)	−1.80 cm/s	−2.71 cm/s	−6.29 cm/s	−2.77 cm/s	−1.83 cm/s	−1.99 cm/s	−2.19 cm/s	−2.19 cm/s	−2.40 cm/s
Druglikeness									
Lipinski	Yes; 1 violation: MLOGP > 4.15	Yes; 1 violation: MLOGP > 4.15	Yes; 0 violation	Yes; 1 violation: MLOGP > 4.15	Yes; 1 violation: MLOGP > 4.15	Yes; 1 violation: MLOGP > 4.15	Yes; 1 violation: MLOGP > 4.15	Yes; 1 violation: MLOGP > 4.15	Yes; 1 violation: MLOGP > 4.15
Ghose	No; 1 violation: WLOGP > 5.6	No; 1 violation: WLOGP > 5.6	Yes	Yes	No; 1 violation: WLOGP > 5.6	No; 1 violation: WLOGP > 5.6	No; 1 violation: WLOGP > 5.6	No; 1 violation: WLOGP > 5.6	No; 1 violation: WLOGP > 5.6
Veber	No; 1 violation: Rotors > 10	No; 1 violation: Rotors > 10	Yes	No; 1 violation: Rotors > 10	No; 1 violation: Rotors > 10	No; 1 violation: Rotors > 10	No; 1 violation: Rotors > 10	No; 1 violation: Rotors > 10	No; 1 violation: Rotors > 10
Egan	No; 1 violation: WLOGP > 5.88	Yes	Yes	Yes	No; 1 violation: WLOGP > 5.88	No; 1 violation: WLOGP > 5.88	No; 1 violation: WLOGP > 5.88	No; 1 violation: WLOGP > 5.88	Yes
Muegge	No; 2 violations: XLOGP3 > 5, Heteroatoms < 2	No; 1 violation: XLOGP3 > 5	Yes	No; 1 violation: XLOGP3 > 5	No; 3 violations: XLOGP3 > 5, Heteroatoms < 2, Rotors > 15	No; 2 violations: XLOGP3 > 5, Rotors > 15	No; 2 violations: XLOGP3 > 5, Rotors > 15	No; 2 violations: XLOGP3 > 5, Rotors > 15	No; 3 violations: MW < 200, XLOGP3 > 5, Heteroatoms < 2
Bioavailability score	0.55	0.55	0.55	0.85	0.55	0.55	0.55	0.85	0.55
Medicinal chemistry									
PAINS	0 alert	0 alert	0 alert	0 alert	0 alert	0 alert	0 alert	0 alert	0 alert
Brenk	0 alert	0 alert	2 alerts: Michael acceptor 1, more than 2 esters	0 alert	0 alert	0 alert	0 alert	0 alert	0 alert
Leadlikeness	No; 3 violations: MW < 250, Rotors > 7, XLOGP3 > 3.5	No; 2 violations: Rotors > 7, XLOGP3 > 3.5	No; 1 violation: MW < 250	No; 2 violations: Rotors > 7, XLOGP3 > 3.5	No; 2 violations: Rotors > 7, XLOGP3 > 3.5	No; 2 violations: Rotors > 7, XLOGP3 > 3.5	No; 2 violations: Rotors > 7, XLOGP3 > 3.5	No; 2 violations: Rotors > 7, XLOGP3 > 3.5	No; 3 violations: MW < 250, Rotors > 7, XLOGP3 > 3.5
Synthetic accessibility	2.26	2.53	2.58	2.31	2.63	2.88	2.76	2.54	2.04
Toxicity (Prediction/ Probability)									
LD50 (mg/kg)	750	5000	5000	900	1000	5000	5000	900	750
Hepatotoxicity	Inactive/0.74	Inactive/0.58	Inactive/0.58	Inactive/0.52	Inactive/0.90	Inactive/0.58	Inactive/0.58	Inactive/0.52	Inactive/0.74
Neurotoxicity	Inactive/0.66	Inactive/0.80	Inactive/0.80	Inactive/0.92	Inactive/0.90	Inactive/0.80	Inactive/0.80	Inactive/0.92	Inactive/0.66
Nephrotoxicity	Inactive/0.87	Inactive/0.59	Active/0.51	Inactive/0.53	Inactive/0.67	Inactive/0.59	Inactive/0.59	Inactive/0.53	Inactive/0.87
Respiratory toxicity	Inactive/1.0	Inactive/0.98	Inactive/0.75	Inactive/0.85	Inactive/0.93	Inactive/0.98	Inactive/0.98	Inactive/0.85	Inactive/1.0
Cardiotoxicity	Inactive/0.83	Inactive/0.99	Inactive/0.79	Inactive/0.99	Inactive/1.0	Inactive/0.99	Inactive/0.99	Inactive/0.99	Inactive/0.83

### In Silico prediction of antiviral and insecticide activity spectra for zoochemicals prediction analysis

**Fig. 4:**
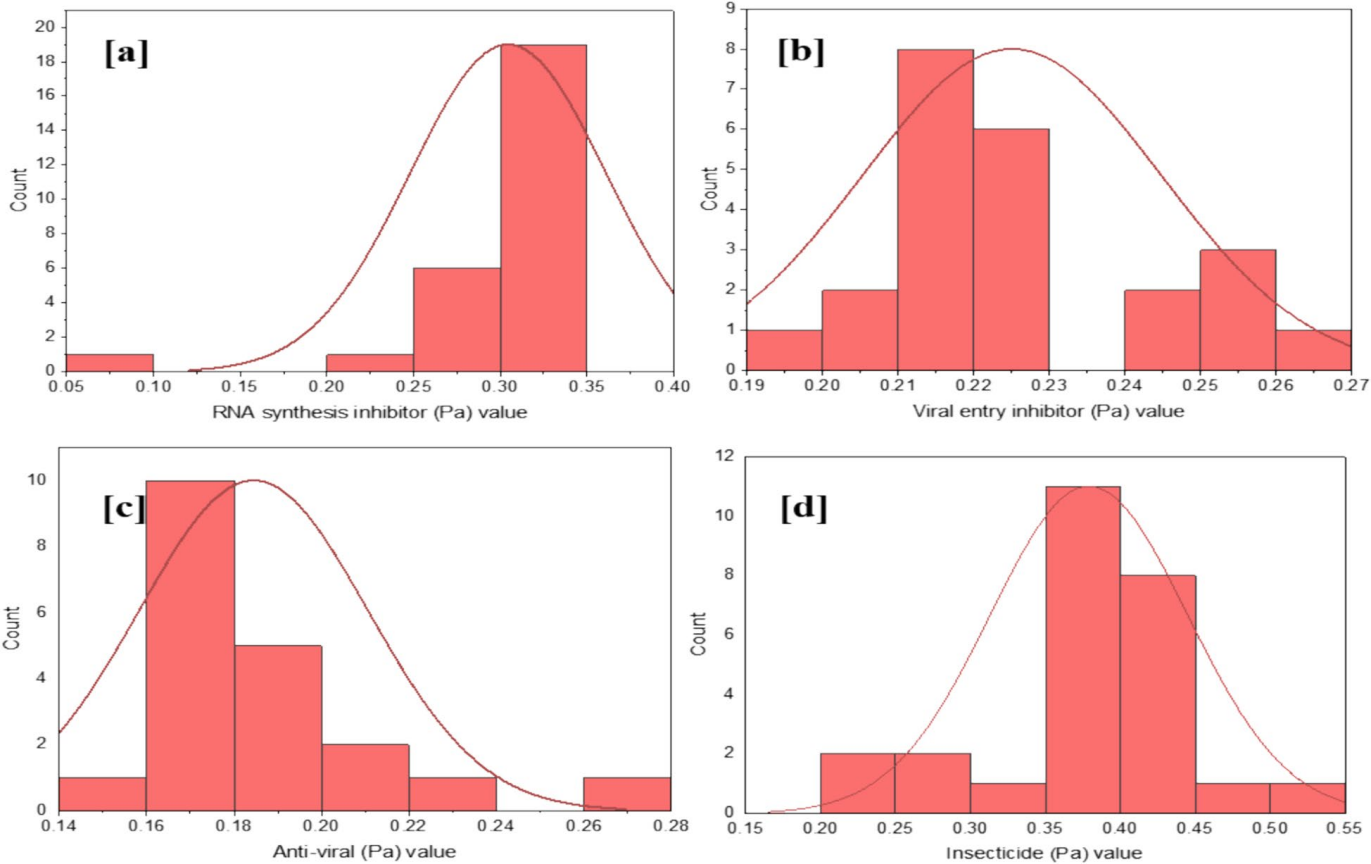
Histogram plot of Pa range (N = 27) of Antiviral and insecticidal activity (**a** RNA synthesis inhibitor; **b** Viral entry inhibitor; **c** Antiviral; **d** Insecticide)

The biological activities of zoochemicals were predicted using the Prediction of Activity Spectra for Substances (PASS) tool, which evaluates the therapeutic potential of bioactive secondary metabolites (Jamkhande et al. 2024; Veerabahu et al. 2024). Compounds with a higher probability of activity (Pa) than probability of inactivity (Pi) are considered pharmacologically promising (Khurana et al. 2011). For *Charybdis natator* shell methanolic extract, Pa values ranged from 0.503 to 0.2 for insecticidal activity, 0.348–0.073 for RNA synthesis inhibition, 0.26–0.196 for viral entry inhibition, and 0.26–0.156 for antiviral activity (Table 6). Notably, Pa values for insecticidal properties were higher than for antiviral effects, suggesting the extract's potential as a natural insecticide and antiviral agent. A histogram (Fig. 4a–d) highlights the distribution of Pa values, underscoring the biological potential of key secondary metabolites in the extract (Jamkhande et al. 2024).

**Table 6:** Anti-viral and Insecticide prediction of *Charybdis natator* shell zoochemicals, using PASS Online predicts (Way2Drug)

Zoochemicals	RNA synthesis inhibitor		Viral entry inhibitor		Anti-viral		Insecticide	
	Pa	Pi	Pa	Pi	Pa	Pi	Pa	Pi
Hexadecane	0.328	0.039	0.259	0.026	–	–	0.378	0.007
Tetradecane	0.328	0.039	0.259	0.026	–	–	0.378	0.007
Heptadecane	0.328	0.039	0.259	0.026	–	–	0.378	0.007
1-Heptadecanol	0.347	0.032	0.212	0.115	0.202	0.094	0.357	0.009
Nonadecanoic acid, methyl ester	0.329	0.039	0.227	0.072	0.176	0.125	0.387	0.007
1-Tridecene	0.266	0.082	0.225	0.076	0.197	0.099	0.435	0.005
9-Octadecenamide, (Z)	0.261	0.088	0.229	0.068	0.186	0.112	0.245	0.026
Dibutyl phthalate	0.270	0.078	0.215	0.104	–	–	0.428	0.005
Diisooctyl phthalate	0.272	0.077	0.212	0.114	0.156	0.155	0.391	0.006
Heptacosanoic acid, methyl ester	0.329	0.039	0.227	0.072	0.176	0.125	0.387	0.007
3,6-Dimethyldecane	0.348	0.032	0.211	0.116	0.260	0.054	0.425	0.005
Phytol	0.336	0.036	–	–	0.239	0.067	0.405	0.006
Octadecanoic acid	0.325	0.040	0.241	0.046	0.186	0.111	0.293	0.018
11-Eicosenoic acid, methyl ester	0.329	0.039	0.200	0.159	0.165	0.141	0.418	0.005
4-Methyl-Z-4-hexadecen-1-ol	0.315	0.045	–	–	0.162	0.146	0.503	0.004
Heptadecanoic acid, heptadecyl ester	0.325	0.040	0.196	0.171	0.162	0.145	0.468	0.004
Dodecane, 4,6-dimethyl	0.348	0.032	0.211	0.116	0.161	0.146	0.369	0.008
Phthalic acid, butyl undecyl ester	0.265	0.084	0.213	0.111	–	–	0.430	0.005
1H-Purin-6-amine, [(2-fluorophenyl)methyl]	0.073	0.036	–	–	0.182	0.117	–	–
1,2-Benzenedicarboxylic acid, diethyl ester	0.293	0.059	0.260	0.024	–	–	0.419	0.005
n-Nonadecanol-1	0.347	0.032	0.212	0.115	0.202	0.094	0.357	0.009
Hexadecanoic acid, methyl ester	0.329	0.039	0.227	0.072	0.176	0.125	0.387	0.007
Octadecanoic acid, methyl ester	0.329	0.039	0.227	0.072	0.176	0.125	0.387	0.007
9-Octadecenoic acid, methyl ester	0.329	0.039	0.200	0.159	0.165	0.141	0.418	0.005
9-Octadecenoic acid,	0.325	0.040	0.213	0.112	0.174	0.128	0.318	0.014
n-Hexadecanoic acid	0.325	0.040	0.241	0.046	0.186	0.111	0.293	0.018
Hexanedioic acid, 2-methyl-5-methylene-, dimethyl ester	0.222	0.137	–	–	–	–	0.200	0.037

### In silico antiviral activity

**Fig. 5:**
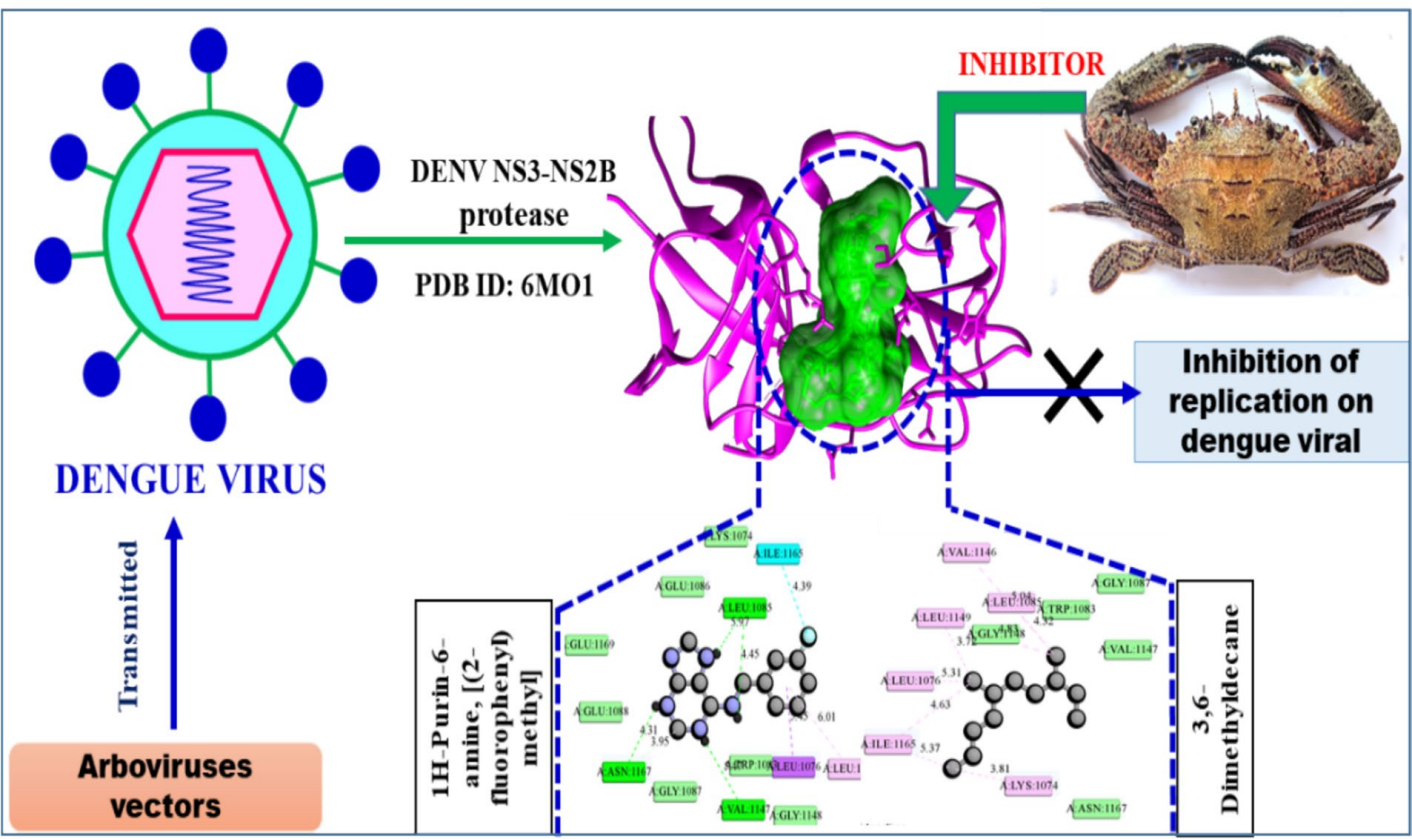
Mode of inhibitory activity of *Charybdis natator* shell zoochemicals against DENV2 NS2B-NS3 (PDB: 6MO1) protease

Dengue and Zika viruses, both flaviviruses and significant human pathogens, are transmitted by mosquito vectors. The highly conserved NS2B-NS3 protease plays a critical role in viral replication, making it an ideal target for rational drug design against these viruses (Yao et al. 2019). Molecular docking studies revealed that zoochemicals exhibit binding affinities (kcal/mol) that effectively inhibit the DENV2 NS2B-NS3 protease (PDB: 6MO1) binding site, indicating potential therapeutic efficacy against dengue virus infection (Table 7, Fig. 5). These findings support the development of zoochemical-based agents targeting the DENV NS2B-NS3 protease to inhibit viral replication (Kronenberger et al. 2021; Adawara et al. 2020).

**Table 7:** In silico anti-viral and larvicidal activity of *Charybdis natator* shell methanolic extract against Arbovirus and Arbovirus vector molecular target using Molecular docking techniques

Zoochemicals	3D Structure	Binding affinity (kcal/mol)			
		Anti-viral molecular target (PDB)			Larvicidal (PDB: 5V13)
		Anti-Dengue (6MO1)	Anti-Zika (5U04)	Anti-Chikungunya (3TRK)	
Hexadecane		−4.50	−4.20	−5.10	−4.70
Tetradecane		−4.40	−4.80	−4.10	−7.00
Heptadecane		−4.40	−4.70	−4.60	−7.40
1-Heptadecanol		−4.90	−4.60	−4.70	−7.30
Nonadecanoic acid, methyl ester		−4.70	−5.00	−4.90	−7.90
1-Tridecene		−4.30	−4.50	−4.20	−6.60
9-Octadecenamide, (Z)		−5.20	−5.30	−5.60	−8.10
Dibutyl phthalate		−5.90	−5.60	−6.40	−8.00
Diisooctyl phthalate		−6.40	−5.40	−6.50	−6.60
Heptacosanoic acid, methyl ester		−4.90	−4.80	−5.20	−7.50
3,6-Dimethyldecane		−5.10	−4.90	−4.70	−4.90
Phytol		−5.10	−5.70	−5.80	−6.00
Octadecanoic acid		−4.90	−4.80	−4.90	−8.00
11-Eicosenoic acid, methyl ester		−5.20	−4.70	−5.40	−5.30
4-Methyl-Z-4-hexadecen-1-ol		−4.70	−5.30	−5.30	−7.80
Heptadecanoic acid, heptadecyl ester		−4.90	−5.30	−5.30	−4.40
Dodecane, 4,6-dimethyl		−4.70	−5.10	−4.90	−7.20
Phthalic acid, butyl undecyl ester		−5.50	−5.90	−6.20	−6.30
1H-Purin-6-amine, [(2-fluorophenyl)methyl]		−7.10	−6.80	−7.20	−8.60
1,2-Benzenedicarboxylic acid, diethyl ester		−6.00	−5.00	−5.60	−6.10
n-Nonadecanol-1		−5.00	−5.10	−4.70	−7.50
Hexadecanoic acid, methyl ester		−5.00	−5.10	−4.90	−5.70
Octadecanoic acid, methyl ester		−4.80	−5.00	−5.00	−6.70
9-Octadecenoic acid, methyl ester		−4.90	−5.30	−5.00	−8.10
9-Octadecenoic acid, (E)		−5.30	−4.80	−5.60	−7.90
n-Hexadecanoic acid		−4.80	−4.40	−4.60	−4.90
Hexanedioic acid, 2-methyl-5-methylene-, dimethyl ester		−5.00	−4.70	−5.00	−6.60

**Fig. 6:**
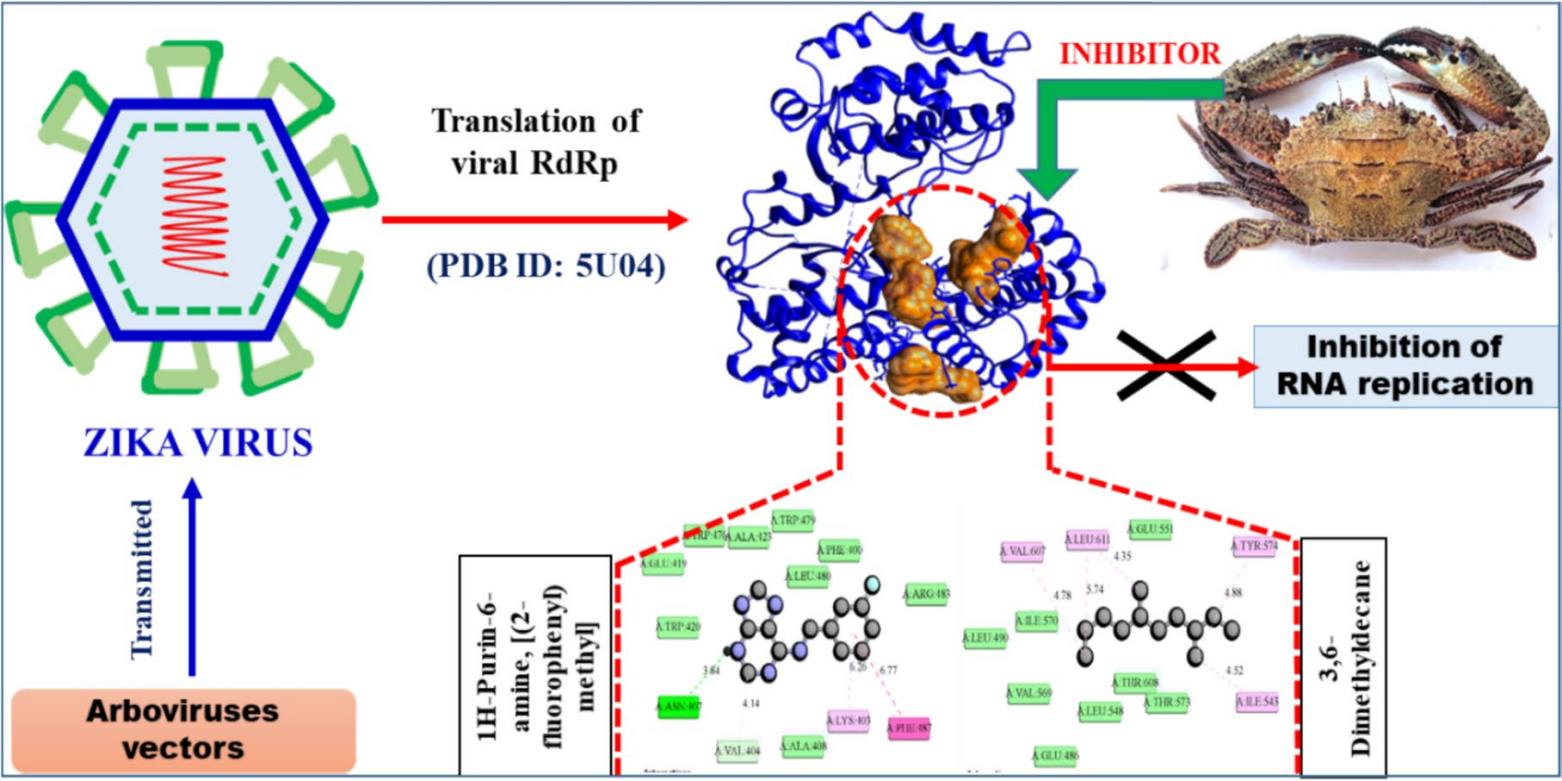
Mode of inhibitory activity of *Charybdis natator* shell zoochemicals against Zika virus ZIKV-NS5-RdRp

The non-structural protein 5 (NS5) RNA-dependent RNA polymerase (RdRp), essential for RNA viral replication, is a prime target for antiviral drug discovery (Godoy et al. 2017). In an in silico anti-Zika virus (ZIKV) study, 28 animal-derived secondary metabolites were screened against the ZIKV NS5 RdRp (PDB: 5U04). The results indicated that *Charybdis natator* shell metabolites could inhibit ZIKV replication, suggesting their potential as anti-ZIKV drug candidates (Table 7 and Fig. 6). These findings highlight the promise of natural bioactive compounds as RdRp inhibitors, with the potential to develop effective anti-ZIKV agents (Chen et al. 2023; Ahmed et al. 2021). Additionally, previous studies support the utility of secondary metabolites in designing inhibitory drugs targeting ZIKV NS5 RdRp (Ho et al. 2023; Buendia-Atencio et al. 2021).

**Fig. 7:**
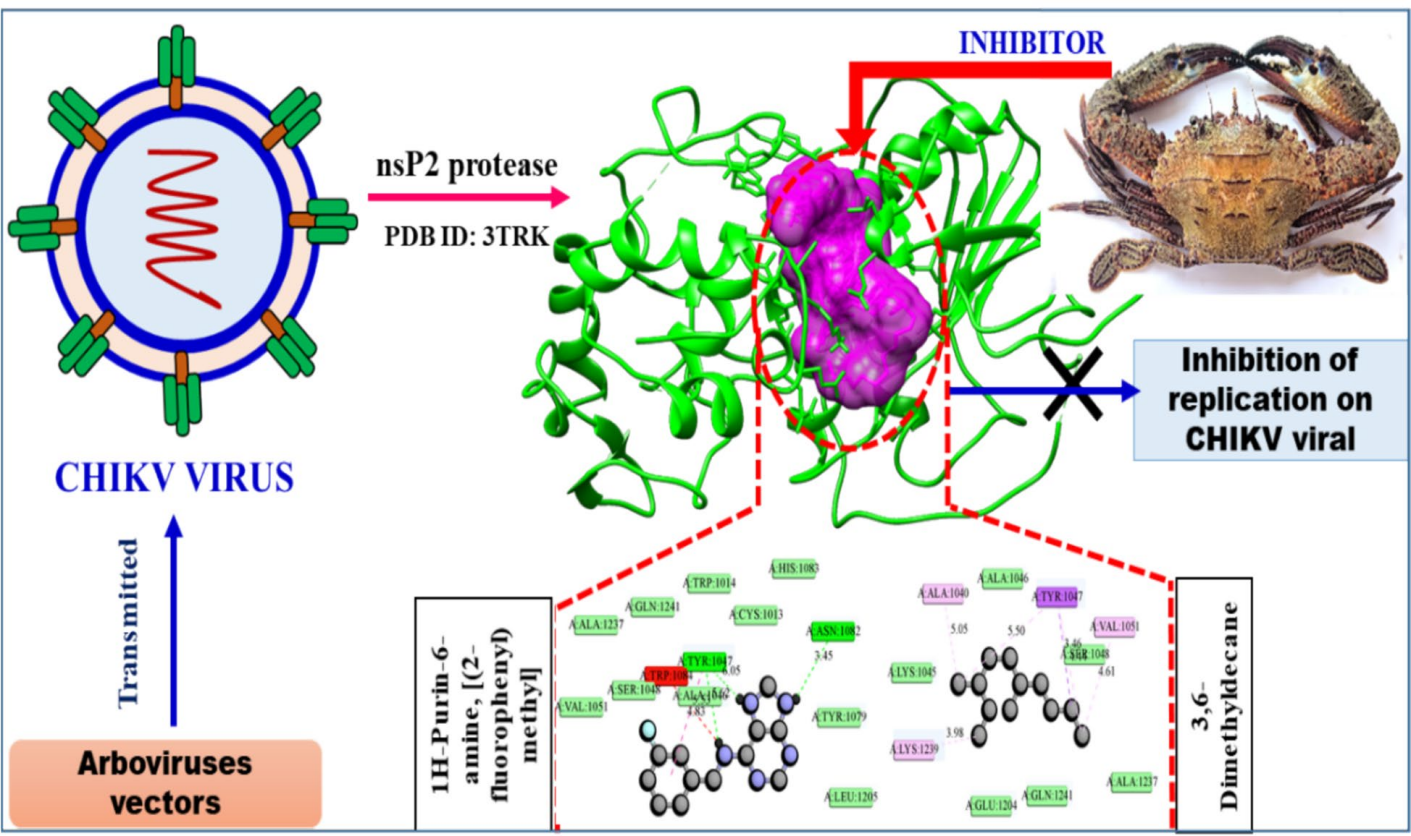
Mode of inhibitory activity of *Charybdis natator* shell zoochemicals against Chikungunya virus nsP2 protease

The nsP2 protein of Chikungunya virus (CHIKV) plays a multifunctional and crucial role in viral replication (Saisawang et al. 2015). As a key target for antiviral drug development, the nsP2 protease has been studied for potential inhibitors using in silico approaches (Nguyen et al. 2015). Table 6 highlights the promising anti-CHIKV activity of *C. natator* shell zoochemicals, specifically their methanolic extract, against the CHIKV nsP2 protease (PDB: 3TRK) (Fig. 7). Additionally, the nsP3 protein emerges as a potential target for therapeutic intervention, suggesting its potential use in treating CHIKV infections (Oo et al. 2016).

Figures 5, 6 and 7 indicate that *C. natator* shell zoochemicals bind to similar sites on multiple antiviral protein targets. In contrast, Fig. 6 reveals three distinct binding sites, suggesting a potential correlation between the number of binding sites and the strength of antiviral activity, particularly against dengue and chikungunya viruses (Table 7). Table 8 shows amino acid binding interactions bond types of the highest active zoochemicals of *Charybdis natator* shell, using PASS and Molecular docking prediction based on antiviral and insecticide properties.

**Table 8:** Amino acid binding interactions bond types of the highest active zoochemicals of *Charybdis natator* shell, using PASS and Molecular docking results

Highest activity zoochemicals (PASS and molecular docking)	Amino acid binding interactions bond types
Anti-dengue (PDB: 6MO1)	
1H-Purin-6-amine, [(2-fluorophenyl)methyl]	Van der waals, Conventional hydrogen bond, Carbon hydrogen bond, Halogen (Fluorine), Pi-Sigma and Pi-Alkyl
3,6-Dimethyldecane	Van der waals and Alkyl
Anti-Zika (PDB: 5U04)	
1H-Purin-6-amine, [(2-fluorophenyl)methyl]	Van der waals, Conventional hydrogen bond, Carbon hydrogen bond, Pi-Pi T-shaped and Pi-Alkyl
3,6-Dimethyldecane	Van der waals, Pi-Alkyl and Alkyl
Anti-Chikungunya (PDB: 3TRK)	
1H-Purin-6-amine, [(2-fluorophenyl)methyl]	Van der waals, Conventional hydrogen bond, Unfavorable donor-donor, Amide-Pi Stacked
3,6-Dimethyldecane	Van der waals, Pi-Sigma, Alkyl, Pi-Alkyl
Larvicidal (PDB: 5V13)	
1H-Purin-6-amine, [(2-fluorophenyl)methyl]	Van der waals, Conventional hydrogen bond, Unfavorable donor-donor, Pi-Alkyl
4-Methyl-Z-4-hexadecen-1-ol	Van der waals, Conventional hydrogen bond, Pi-Sigma, Alkyl and Pi-Alkyl

**Fig. 8:**
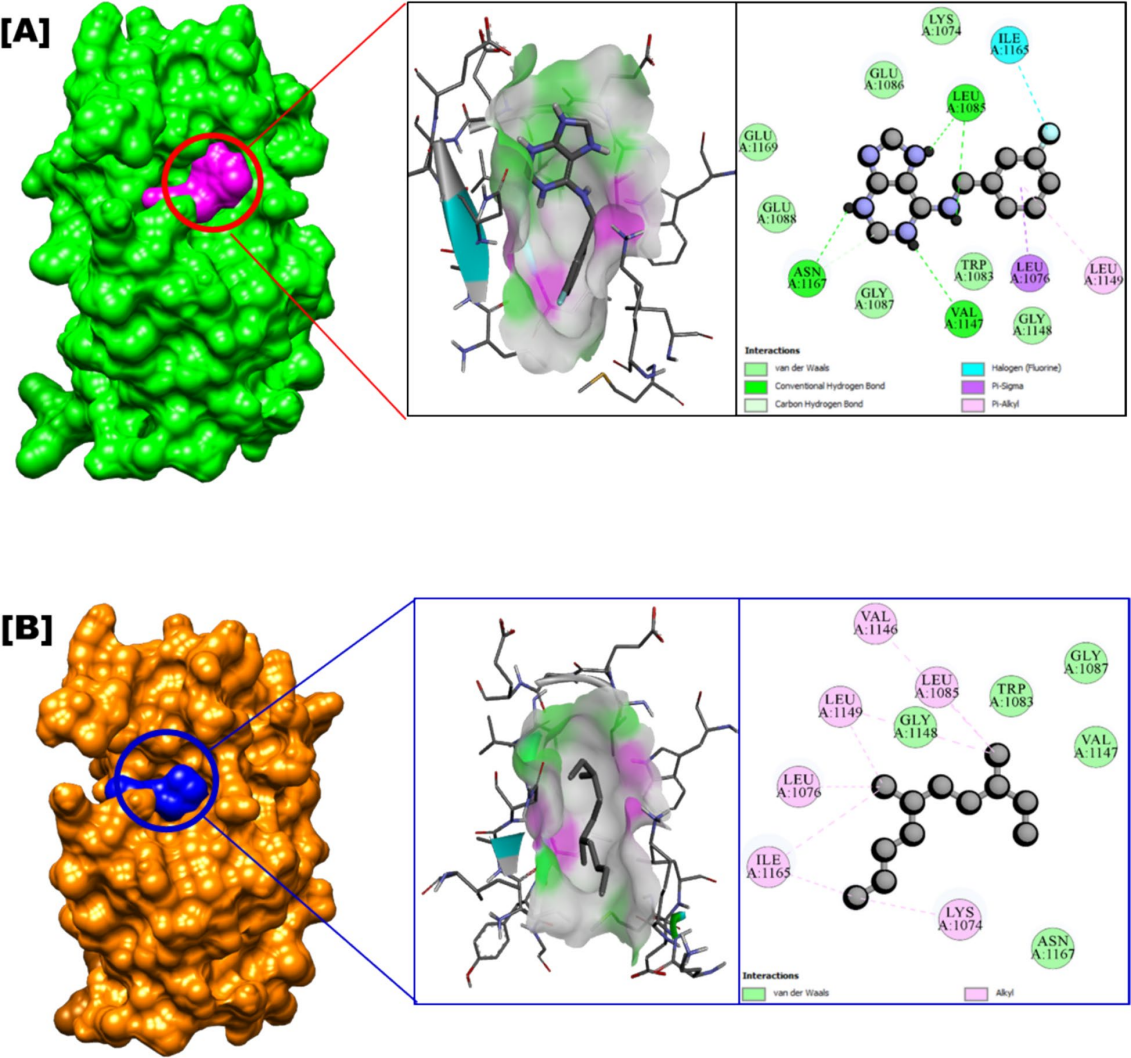
**A** 3D Docked complex and 2D interaction of molecular docking highest Anti-dengue activity of 1H-Purin-6-amine, [(2-fluorophenyl)methyl]. **B** 3D Docked complex and 2D interaction of highest RNA synthesis inhibitor Pa value (3,6-Dimethyldecane) with Anti-dengue target molecular docking

**Fig. 9:**
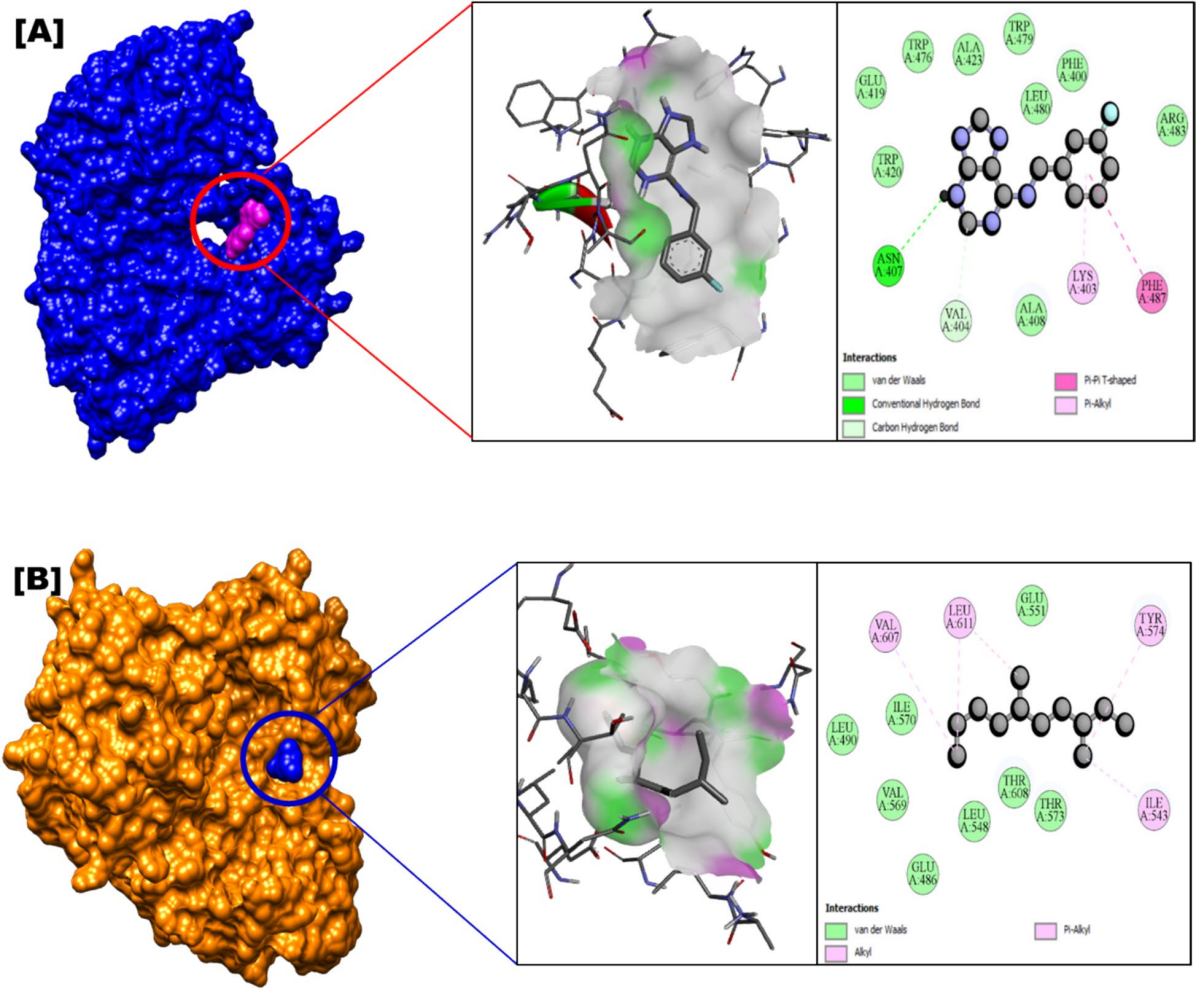
**A** 3D Docked complex and 2D interaction of molecular docking highest Anti-zika activity of 1H-Purin-6-amine, [(2-fluorophenyl)methyl]. **B** 3D Docked complex and 2D interaction of highest RNA synthesis inhibitor Pa value (3,6-Dimethyldecane) with Anti-zika target molecular docking

**Fig. 10:**
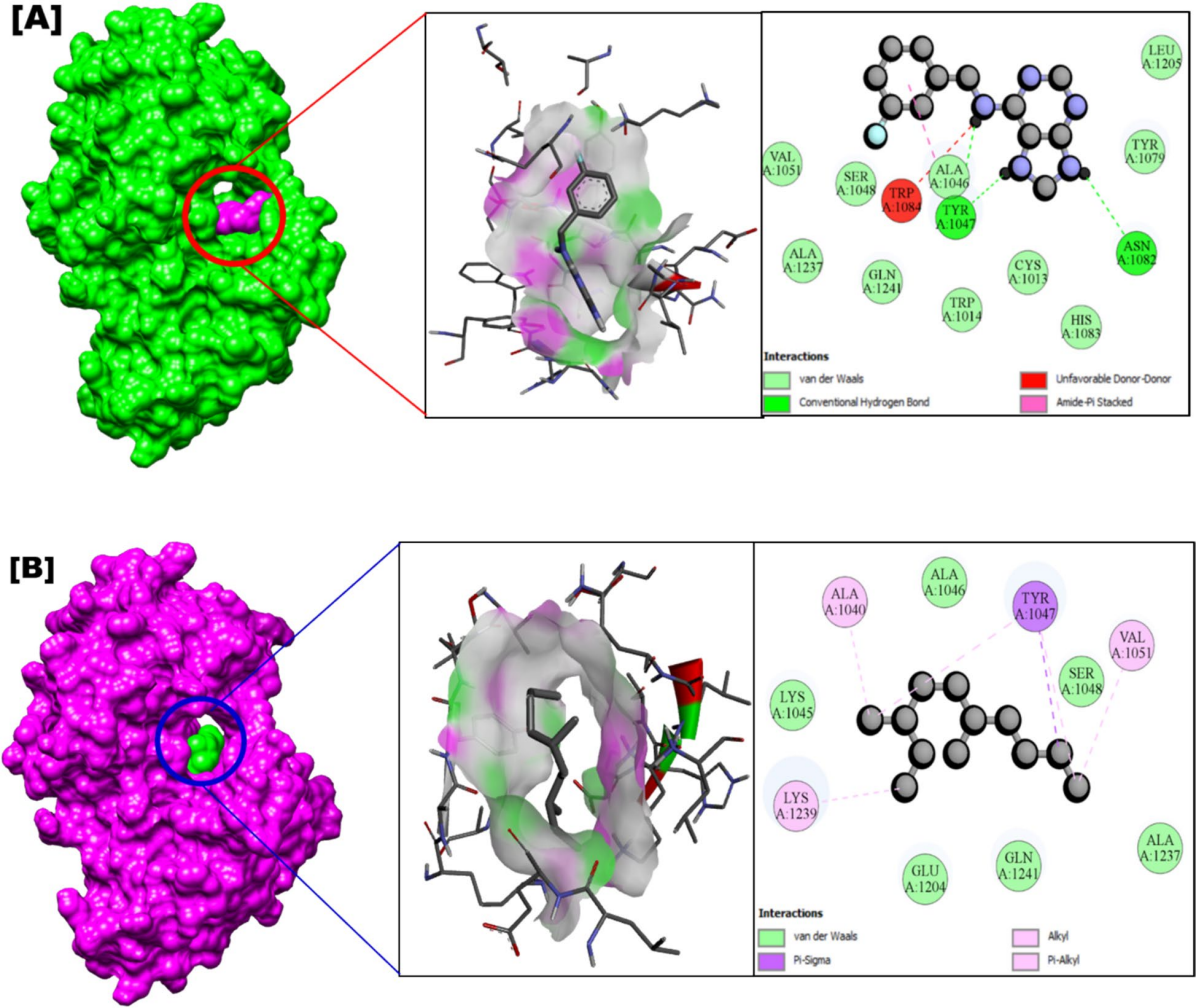
**A** 3D Docked complex and 2D interaction of molecular docking highest Anti-Chikungunya activity of 1H-Purin-6-amine, [(2-fluorophenyl)methyl]. **B** 3D Docked complex and 2D interaction of highest RNA synthesis inhibitor Pa value (3,6-Dimethyldecane) with Anti-Chikungunya target molecular docking

**Fig. 11:**
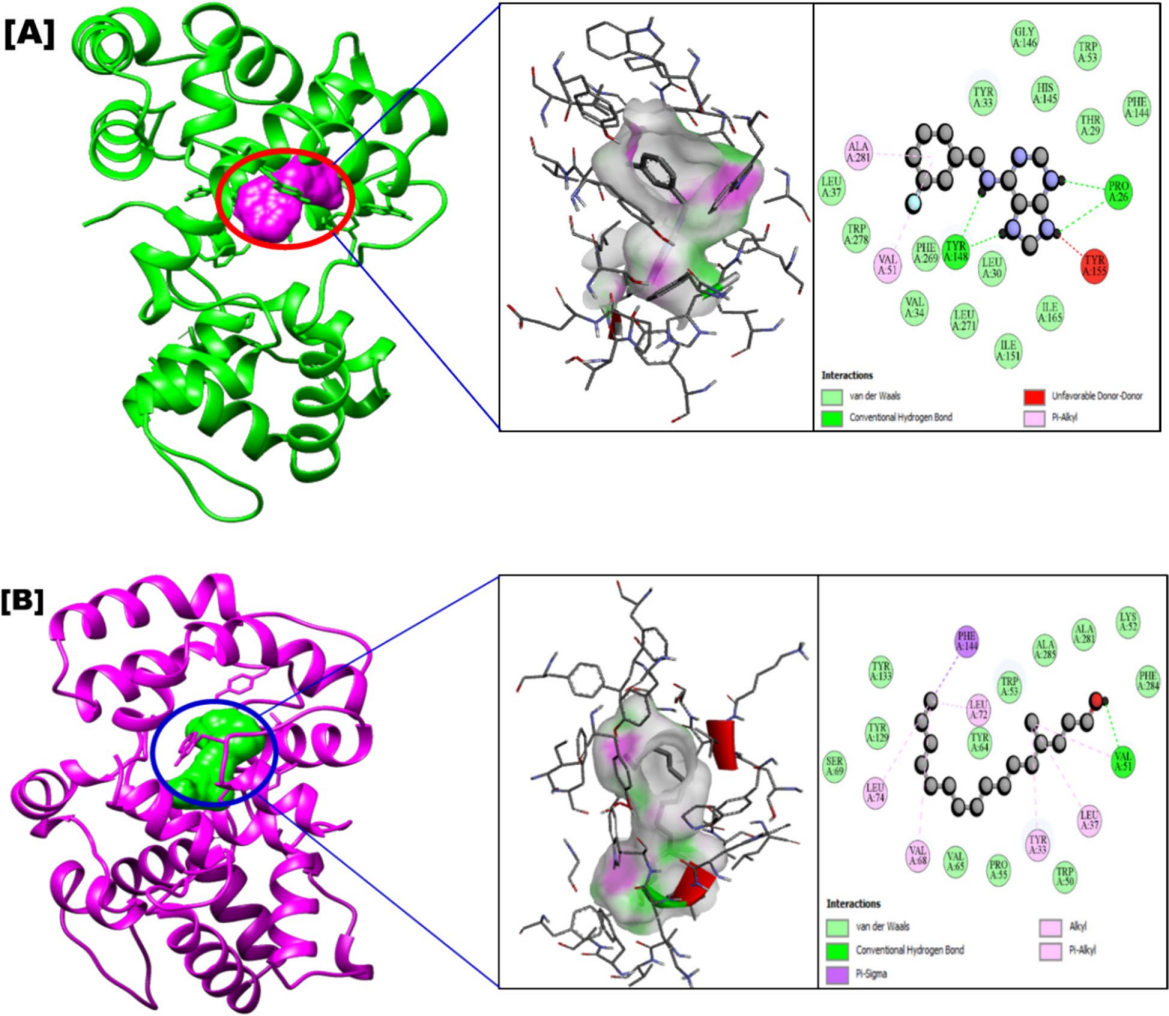
**A** 3D Docked complex and 2D interaction of molecular docking highest larvicidal activity of 1H-Purin-6-amine, [(2-fluorophenyl)methyl]. **B** 3D Docked complex and 2D interaction of highest Insecticide Pa value (4-Methyl-Z-4-hexadecen-1-ol) with larvicidal target molecular docking

Table 9 presents a correlation analysis of the binding affinities of 27 zoochemicals from *Charybdis natator* shells to key antiviral protein targets. A strong positive correlation was observed between the inhibitory activities against dengue, Zika, and chikungunya viruses (r = 0.726, 0.889, and 0.789, respectively). These findings suggest a shared mechanism of action for these zoochemicals, making them promising candidates for the development of cost-effective, broad-spectrum antiviral agents against arboviruses like dengue, Zika, and chikungunya. Among the zoochemicals derived from *Charybdis natator* shells, 1H-Purin-6-amine and [(2-fluorophenyl)methyl] exhibited the highest activity to multiple molecular targets. 3,6-Dimethyldecane showed the highest potential activity as an RNA synthesis inhibitor and antiviral agent (Figs. 8, 9, 10a, b, while 4-Methyl-Z-4-hexadecen-1-ol demonstrated the strongest insecticidal potential compared to other zoochemicals (Fig. 11a, b).

**Table 9:** Correlation matrix of in silico anti-viral molecular docking of zoochemicals (N = 27) against Arbovirus molecular target

Molecular target	Anti-Dengue (6MO1)	Anti-Zika (5U04)	Anti-Chikungunya (3TRK)
Anti-Dengue (6MO1)	1		
Anti-Zika (5U04)	0.726	1	
Anti-Chikungunya (3TRK)	0.889	0.789	1

**Fig. 12:**
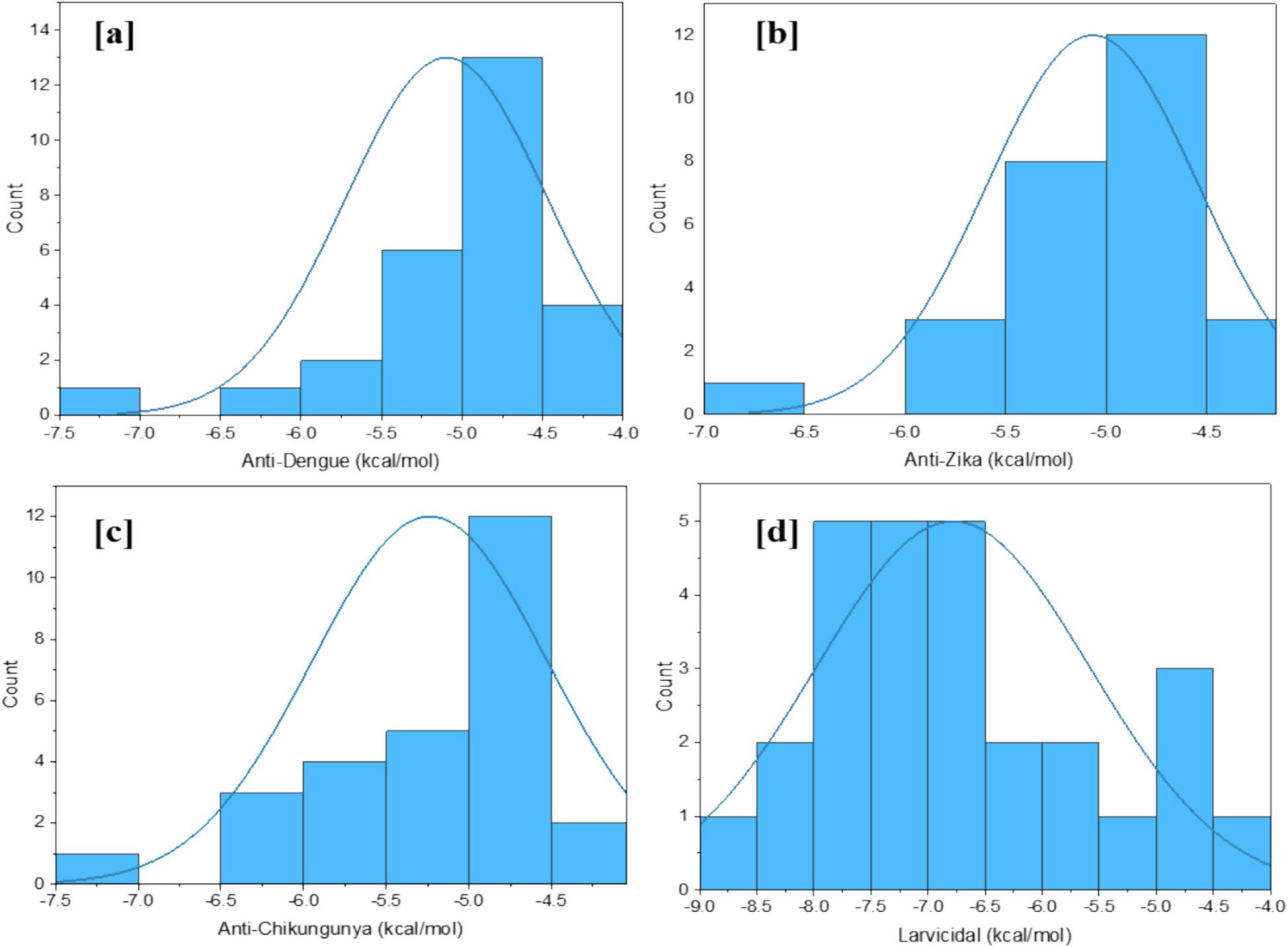
**a**, **b** Histogram plot of antiviral target (**a** Anti-Dengue; **b** Anti-Zika; **c** Anti Chikungunya) and larvicidal target (**d** Larvicidal) binding affinity of *C. natator* shell zoochemicals (n = 27)

Figure 12a–d demonstrate that the highest binding affinity range for antiviral activity (−5.0 to −4.5 kcal/mol) is observed across all target proteins. This suggests that *C. natator* shell zoochemicals within this affinity range have the strongest potential for antiviral efficacy. Notably, these zoochemicals exhibit the highest efficacy against the chikungunya virus (PDB: 3TRK), followed by dengue (PDB: 6MO1) and Zika (PDB: 5U04). Figure 12d shows that *C. natator* shell zoochemicals exhibit a high binding affinity range (−8.0 to −6.5 kcal/mol) to the larvicidal target, indicating their potential to control mosquito larvae growth.

### Larvicidal activity of marine crab *Charybdis natator* shell methanolic zoo-extract against *Aedes aegypti* larvae

**Fig. 13:**
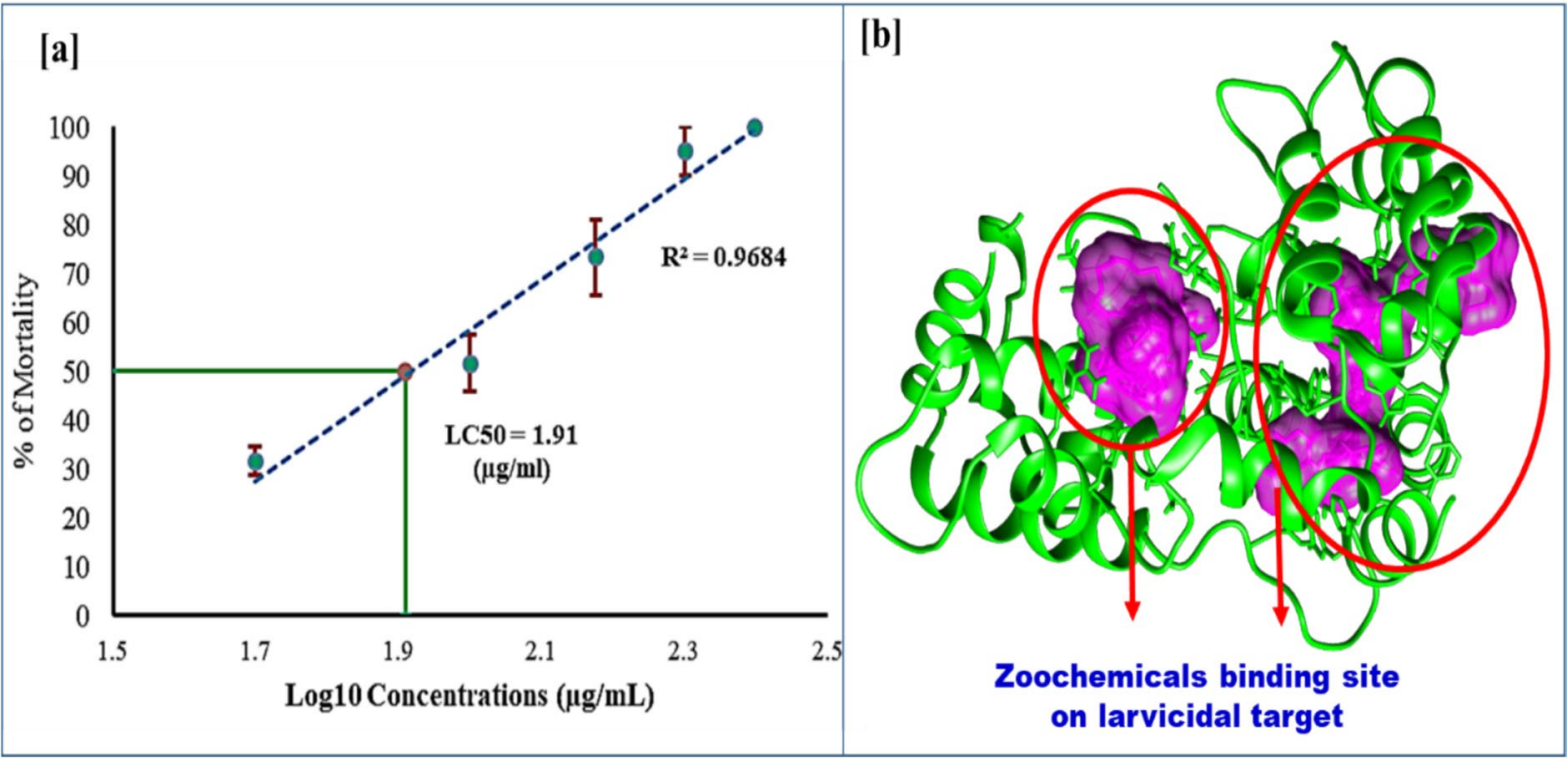
Larvicidal activity of *Charybdis natator* shell against *Aedes aegypti* 4^th^ instar larvae. **a** Larvicidal bioassay, and **b** In silico approach)

Mosquitoes, particularly *Aedes* species, are significant vectors of deadly arboviruses such as dengue, Zika, and chikungunya. To address this global health issue, researchers have explored natural alternatives to synthetic insecticides. A methanolic extract of *Charybdis natator* crab shells has demonstrated potent larvicidal activity against *Aedes aegypti* fourth-instar larvae, with an LC_50_ value of 81.001 µg/mL. This dose-dependent effect (R^2^ = 0.968) highlights the potential of this extract as a safe and effective zoo-insecticide (Table 10 and Fig. 13a, b). Further in silico molecular docking studies have confirmed the larvicidal activity of *C. natator* shell zoochemicals against mosquito juvenile hormone binding protein (Table 7). Figure 13 illustrates the mode of inhibitory action of these compounds, revealing their potential to disrupt mosquito development and reproduction.

**Table 10:** Larvicidal activity of *Charybdis natator* shell against *Aedes aegypti* 4th instar larvae

Zoo-extract	Lethal concentration	LCL	UCL	R^2^	Chi-Square
LC50 (µg/mL)	81.001	59.778	99.439	0.968	4.010 (*p* = 0.260^a^; df = 3)
LC90 (µg/mL)	193.241	152.530	294.461		

LCL: 95% Lower Confidence Limits; UCL: 95% Upper Confidence Limits; a: Since the significance level is greater than 0.050, no heterogeneity factor is used in the calculation of confidence limits

Insect metamorphosis, the transformation from larvae to adults, is regulated by hormones such as juvenile hormone (Lv et al. 2024; Malhotra and Basu 2023). By targeting this hormone, researchers aim to develop novel zoo-insecticides to control mosquito populations. Chitosan nanoparticles derived from crab and mantis shrimp shells have demonstrated larvicidal activity against *Aedes aegypti* third-instar larvae. These nanoparticles offer a promising eco-friendly approach to mosquito control (Anand et al. 2014). Additionally, ethanolic extracts from *Allacanthos* crabs have exhibited anti-inflammatory and antioxidant properties. These extracts have shown significant cytotoxicity against human liver cancer (HepG2) cells, suggesting their potential as a source of anticancer agents (Rehman et al. 2020).

**Fig. 14:**
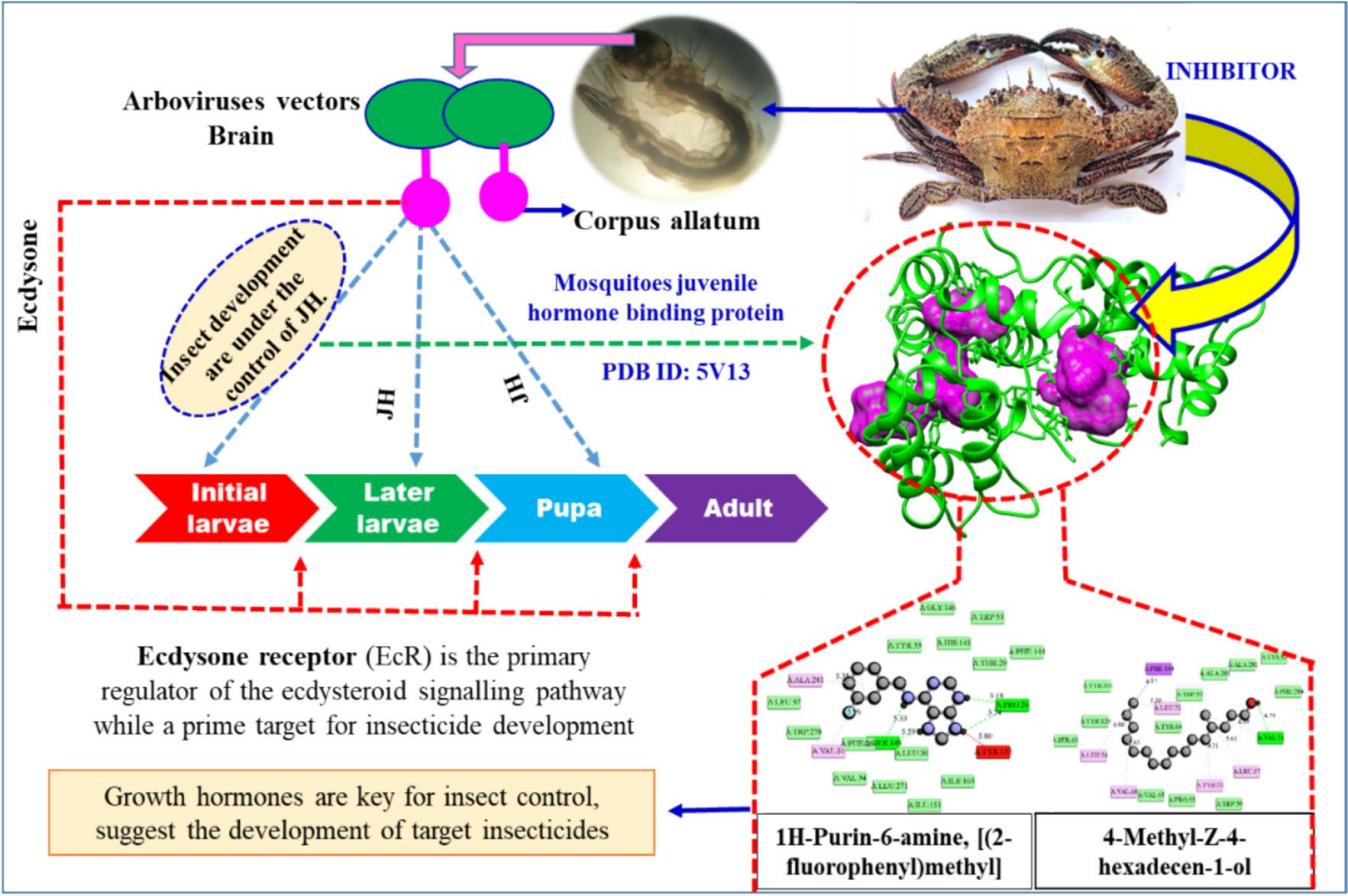
Mode of inhibitory activity of *Charybdis natator* shell zoochemicals against *Aedes aegypti* Mosquitoes juvenile hormone binding protein

Bioactive compounds derived from *C. natator* shells have the potential to combat oxidative stress and inflammation, which are implicated in numerous diseases. Terpenes and fatty acid derivatives are among the most common classes of metabolites found in these shells, many of which possess larvicidal properties. Crabs like *Portunus pelagicus* and *Scylla tranquebarica* are known to produce secondary metabolites with antibacterial activity, suggesting their potential for pharmaceutical applications (Galal-Khallaf et al. 2024; Lim et al. 2023; Laith et al. 2017). Similarly, *C. natator* shell zoochemicals, classified as secondary metabolites, have shown inhibitory activity against juvenile hormone binding proteins (PDB: 5V13: Fig. 14). By targeting this key protein, these compounds can disrupt *Aedes aegypti* larval development, ultimately reducing the transmission of arboviral diseases.

## Conclusion

The marine crab *Charybdis natator* shell is a rich source of fatty acid derivatives, including 9-octadecenamide, diisooctyl phthalate, and various fatty acids. These compounds exhibit potent antiviral and larvicidal properties. Methanolic extracts from *C. natator* shells have been shown to effectively control mosquito growth and suppress viral infection and replication. Computational studies further support the potential of these extracts to reduce the transmission of arboviruses such as dengue, Zika, and chikungunya. Statistical analysis of antiviral target correlations indicates a similar mode of action for *C. natator* shell compounds against different arboviruses. Importantly, these compounds demonstrate comparable efficacy in suppressing the replication of each virus. Given their biodegradability and effectiveness, *C. natator* shell-derived compounds hold significant promise as eco-friendly therapeutic agents for the treatment of viral infections.

## Data Availability

All data used to support the finding of this study are available from the corresponding authored upon request.

## Abbreviations

CHIKV: Chikungunya
DENV: Dengue
FTIR: Fourier transform infrared
GC–MS: Gas chromatography-mass spectrometry
HepG2: Human liver cancer
JE: Japanese Encephalitis
PASS: Prediction of activity spectra for substances
PDB: Protein data bank
SPSS: Statistical package for the Social Sciences
WNV: West Nile
ZIKV: Zika
